# Effectiveness of early vocational rehabilitation versus usual care to support RETurn to work after stroKE: A pragmatic, parallel-arm multicenter, randomized controlled trial

**DOI:** 10.1177/17474930241306693

**Published:** 2024-12-31

**Authors:** KA Radford, A Wright-Hughes, E Thompson, DJ Clarke, J Phillips, J Holmes, K Powers, D Trusson, K Craven, C Watkins, A Bowen, C McKevitt, J Stevens, JD Murray, RJ O’Connor, S Pyne, H Risebro, R Cameron, TH Sach, F Day, AJ Farrin

**Affiliations:** 1 Centre for Rehabilitation and Ageing Research, School of Medicine, Medical School Queen’s Medical Centre, Nottingham, UK; 2 Clinical Trials Research Unit, Leeds Institute for Clinical Trials Research, University of Leeds, Leeds, UK; 3 Academic Unit for Ageing and Stroke Research, Leeds Institute of Health Sciences, University of Leeds, Leeds, UK; 4 Applied Health Research hub and School of Nursing and Midwifery, University of Central Lancashire, Preston, UK; 5 Manchester Centre for Health Psychology, Geoffrey Jefferson Brain Research Centre, Division of Psychology & Mental Health, University of Manchester, Manchester, UK; 6 Division of Health and Social Care Research, King’s College London, London, UK; 7 PPI, UK; 8 Different Strokes, London, UK; 9 Academic Department of Rehabilitation Medicine, University of Leeds, Leeds, UK; 10 Norwich Medical School, University of East Anglia, Norwich, UK; 11 School of Primary Care, Population Sciences and Medical Education, University of Southampton, Southampton, UK

**Keywords:** Stroke, work, rehabilitation, occupational therapy, randomized controlled trial

## Abstract

**Background:**

Return-to-work is a major goal achieved by fewer than 50% stroke survivors. Evidence on how to support return-to-work is lacking.

**Aims:**

This study aimed to evaluate the clinical effectiveness of Early Stroke Specialist Vocational Rehabilitation (ESSVR) plus usual care (UC) (i.e. usual NHS rehabilitation) versus UC alone for helping people return-to-work after stroke.

**Methods:**

This pragmatic, multicentre, individually randomized controlled trial with embedded economic and process evaluations compared ESSVR with UC in 21 NHS stroke services across England and Wales. Eligible participants were aged ⩾ 18 years, in work at stroke onset, hospitalized with new stroke and within 12 weeks of stroke. People not intending to return-to-work were excluded. Participants were randomized (5:4) to individually tailored ESSVR delivered by stroke specialist occupational therapists for up to 12 months or usual National Health Service rehabilitation. Primary outcome was self-reported return-to-work for ⩾ 2 h per week at 12 months. Primary and safety analyses were done in the intention-to-treat population.

**Results:**

Between 1 June 2018, and 7 March 2022, 583 participants (M_age_ 54.1 years (SD 11.0), 69% male) were randomized to ESSVR (n = 324) or UC (n = 259). Primary outcome data were available for 454 (77.9%) participants. Intention-to-treat analysis showed no evidence of a difference in the proportion of participants returned-to-work at 12 months (165/257 (64.2%) ESSVR vs 117/197 (59.4%) UC; adjusted odds ratio 1.12 (95% CI: 0.75–1.68), p = 0.5678). There was some indication that older participants and those with more post-stroke impairment were more likely to benefit from ESSVR (interaction p = 0.0239 and p = 0.0959, respectively).

**Conclusion:**

To our knowledge, this is the largest trial of a stroke vocational rehabilitation (VR) intervention ever conducted. We found no evidence that ESSVR conferred any benefits over UC in improving return-to-work rates 12 months post-stroke. Return-to-work (for at least 2 h per week) rates were higher than in previous studies (64.2% ESSVR vs 59.4% UC) at 12 months and more than double that observed in our feasibility trial (26%). Interpretation of findings was limited by a predominantly mild–moderate sample of participants and the COVID-19 pandemic. The pandemic impacted the trial, ESSVR and UC delivery, altering the work environment and employer behavior. These changes influenced our primary outcome and the meaning of work in people’s lives; all pivotal to the context of ESSVR delivery and its mechanisms of action.

**Data access:**

Data available on reasonable request.

**Registration:**

ISRCTN12464275.

## Introduction

In the United Kingdom, stroke occurs in more than 100,000 people per year,^1^ with increasing incidence among working-age people^2^ and stroke-related productivity losses estimated to reach £2.1 billion by 2025.^3^ Although reported rates vary, only approximately half UK stroke survivors return-to-work by 1 year.^4,5^ Work is a human right and central to identity providing income, and a sense of purpose.^6^ Good work is protective of health, well-being, and longevity.^7,8^

Government policy and clinical guidelines^9–11^ recognize the need to support stroke survivors of all ages to return-to-work. Vocational rehabilitation (VR) enables people who develop health conditions to overcome obstacles to accessing, maintaining, or returning-to-work or other meaningful occupation.^12^ However, there is little evidence of the effectiveness of post-stroke VR interventions.^13^ A single South African trial (n = 80) of a 6-week occupational therapist (OT) and physiotherapist workplace intervention, reported more intervention participants returned-to-work (60%) at 6-months post-stroke than usual care (UC) (20%).^14^ Our single-center feasibility trial in 46 stroke survivors found that Early Stroke Specialist Vocational Rehabilitation (ESSVR) could be delivered in people with a range of post-stroke disability (37% moderate or moderate–severe stroke),^15,16^ with 39% versus 26% of controls returned-to-work at 12 months (paid/unpaid ⩾ 1-h per week or full-time education).

## Aims

We conducted the RETurn-to-work After stroKE (RETAKE) trial to test the clinical effectiveness of ESSVR on stroke survivors’ return-to-work at 12 months.

## Methods

### Study design and participants

RETAKE was a pragmatic, multicentre, researcher-blinded, individually randomized controlled, partially nested, superiority trial of occupational-therapy-led ESSVR plus UC versus UC alone conducted in 21 English and Welsh NHS stroke services.^17^ An eight-site internal pilot assessed recruitment after 6 months and follow-up after another 6 months. An embedded cost-effectiveness^18^ and process evaluation are reported separately.^19–24^ Patient and public involvement (PPI) throughout provided valuable contributions to trial design, documentation, progress, and outputs. The methods have been reported in detail elsewhere^17,25^ and undertaken after appropriate NHS ethical approval (East Midlands—Nottingham 2 Research Ethics Committee Ref: 18/EM/0019).

Eligible participants were adults (⩾ 18 years of age), admitted to hospital with new stroke and in work (paid/unpaid ⩾ 2 h per week) at stroke onset. Those not intending to return-to-work were excluded. Nominated and eligible carers (main informal caregiver, providing support once or more per week) could join the study. Stroke survivors and carers had to be willing and with capacity to provide informed consent to participate in the study, and sufficient English to contribute to data collection. Written informed consent was required, or verbal consent observed by an independent witness if unable to sign their name or mark the consent form.

Stroke services were eligible if they had capacity to deliver ESSVR and were not routinely providing well-defined VR within 12 weeks of stroke. OTs experienced in delivering specialist stroke rehabilitation in community settings were preferred.

### Randomization and masking

Participants were randomly assigned to ESSVR or UC sequentially, with 5:4 allocation ratio to account for the partially nested study design (participants nested within OTs in ESSVR). Allocation was via a computer-generated minimization program incorporating random element, stratified by site, participant age (< 55, ⩾ 55 years) and stroke severity (derived from EQ-5D-5L mobility question, picture naming, and executive tasks from the Oxford Cognitive Screen (OCS)).^26^ Blinding of participants and OTs was not possible. Researchers were masked to allocation.

### Procedures

Following admission into a stroke service, screening, informed consent, and baseline assessments will be completed within 12 weeks of stroke onset, prior to randomization and allocation.

ESSVR was developed according to the Medical Research Council (MRC) framework for complex interventions^24,27^ and underwent prior feasibility testing.^15,16^ ESSVR was delivered by specially trained RETAKE OTs using a case-coordination model of early intervention VR up to 12 months post-randomization. ESSVR was originally designed for in-person delivery at the participants home, work or in the community, later adapted to remote delivery because of the pandemic. ESSVR was individually tailored according to the participants’ needs, preferences, and employment context; it included assessing the impact of stroke on the job, educating patients and employers about stroke impact, work preparation, and liaison with employers. RETAKE OTs training, intervention delivery, mentoring, and Competency assessment are described elsewhere.^20–23,28,29^ UC was offered to participants in both trial arms according to site’s available routine rehabilitation services. RETAKE OTs could not provide treatment to UC participants to prevent contamination. UC data were self-reported using participant questionnaires.

Researchers collected baseline demographics, details of stroke, and the OCS^26^ to assess major cognitive domains. Questionnaires capturing patient- and carer-reported measures were administered by post or online at baseline and 3-, 6-, and 12-month post-randomization. Priming calls, reminder letters/emails, and SMS text message prompts supported data return. Two-way SMS text messages were sent to non-responders to confirm return-to-work only (the primary outcome), followed by a telephone call or face-to-face home visit. Primary 12-month return-to-work outcome data were collected retrospectively from non-responders latterly in the overall trial follow-up period. We intended to obtain aggregated work status via routine data transfers from the Department for Work and Pensions (DWP).

### Outcomes

The primary outcome was self-reported return-to-work status at 12-month post-randomization. “In” work, meant participants were in paid or unpaid work (including pre-stroke, new, or adapted roles) for at least 2 h per week.

Secondary outcomes, participant self-reported at 3-, 6- and 12-month post-randomization (unless stated otherwise), included:

- return-to-work at 3 and 6 months;
- changes in role, hours worked per week, and days in work following return-to-work;
- mood (Hospital Anxiety and Depression Scale (HADS));^30^
- functional ability (Nottingham Extended Activities of Daily Living (NEADL));^31^
- social participation (Community Integration Questionnaire (CIQ) social and productivity scores)^32^ at 12 months;
- work self-efficacy (single question from the work ability index (WAI));^33^
- confidence (Confidence After Stroke Measure (CASM))^34^ at 12 months;
- carer burden (Modified Caregiver Strain Index (MSCI)).^35^

Adverse events included death (reported by site), hospital attendances, and work accidents (participant self-report).

### Usual care

Our approach to understanding UC in the context of this trial was threefold and described elsewhere;^25^ (1) Self-reported resource use data were collected from participants at each follow-up, (2) an embedded case study design and for a randomly selected 5% of participants in both arms involving repeated (a) observation of intervention delivered and (b) interviews with participants, treating therapists’ and participants’ employers (where permitted), (c) extracted detail from UC therapy records, SNAPP data, and participants’ self-reported resource use to establish a “complete” picture, (3) survey of participating sites pre- and post-recruitment to understand UC pathways and VR service developments in the trial lifetime.

### Statistical analysis

We estimated 760 participants (420 ESSVR, 340 UC) would provide 90% power with two-sided 5% significance level to detect a 13% absolute difference in the proportion of people meeting the primary outcome, allowing for 20% loss to follow-up. This assumed 26% return-to-work in UC as per our feasibility study^15^ and an average cluster size of 11 ESSVR participants per OT (0.68 coefficient of variation), 0.03 intracluster correlation. Due to the pandemic, the sample size target was reduced to 582 participants (308 ESSVR, 274 UC) to provide 80% power, with updated average cluster size assumption of seven participants per OT.

We analyzed effectiveness outcomes according to the intention-to-treat population, defined as all participants randomly allocated, regardless of adherence. All statistical testing used two-sided 5% significance levels and were conducted in SASv9.4. We undertook single final analysis of outcome data (including internal pilot data) with no interim analyses.

We analyzed the primary outcome using a generalized logistic mixed-effects partially nested regression model,^36^ adjusted for site, age, gender, mobility, OCS picture naming (aphasia) and OCS executive mixed scores (cognition) as fixed effects, and OT random effect (see Supplementary materials), to test for differences between treatment groups on 12-month return-to-work status. We analyzed secondary outcomes similarly using logistic or linear regression adjusted for respective baseline score, as appropriate. Results were expressed as adjusted odds ratios (OR, ESSVR/UC) or mean differences (MD, ESSVR/UC), together with 95% CIs and p-values. Assumptions were checked for all regression models using residual plots. Missing data were imputed by treatment group via multiple imputation by chained equations with 50 imputations, including fixed covariates, variables predictive of missingness, and outcome at preceding timepoints (see Supplementary materials). Results of identical analyses performed on each of the imputed datasets were combined using Rubin’s rules. Sensitivity analyses used complete data.

Prespecified exploratory moderator analyses of the primary outcome investigated whether the treatment effect varied by covariates, number of impairments, role, pre-stroke working hours, recruitment period, and baseline questionnaire scores, by including a treatment–moderator interaction in the primary analysis model. Further exploratory analysis explored the impact of participant intervention adherence using complete data in a complier average causal effect analysis and by excluding non-compliers.

## Results

**Figure 1.**
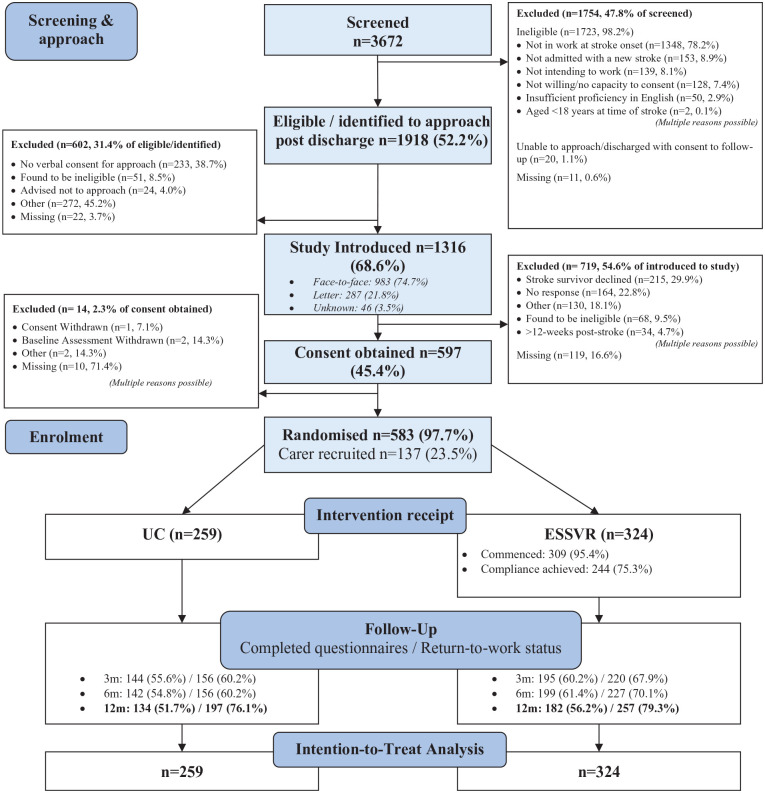
CONSORT Diagram.

Between 1 June 2018, and 7 March 2022, 3672 patients were screened, and 583 participants randomly assigned to ESSVR (n = 324) and UC (n = 259) (Figure 1). Carers were recruited for 137 (23.5%) participants. Due to the pandemic, recruitment was paused 31 March to 1 August 2020. Most participants were recruited pre-COVID (76.3%), but the trial completed for only 28.5%; 12.3% were recruited during and 11.3% after the UK Coronavirus Job Retention (furlough) scheme applied.^37^ The impact of COVID on trial participants is summarized in Tables S7–8.

Baseline characteristics were balanced across arms (Table 1, Table S1–3). Participants were mostly male (400, 69.0%), White (453, 83.7%), with mean age 54 years (SD 11.1); compared to 52.1% male, mean age 64.2 years (SD 15.8) screened (Table S1). Participants were well educated (41.7% higher education, i.e. university degree or equivalent) and worked in an equal mix of blue- and white-collar roles. Participants were mostly ischemic stroke survivors (82.8%), recruited a median 28 days post-stroke (IQR 13–44) having spent a median 4 days in hospital (IQR 2–10). Half had no pre-stroke comorbidities known to affect work. Half had no or mild post-stroke impairments in mobility (EQ-5D-5L indicated no/only slight problems walking), cognition (OCS executive mixed task score ⩽ 4/13), or expressive language (OCS picture naming task score ⩽ 3/4) and only 10.6% had more than one of these impairments, indicative of a mostly mild–moderate severity sample.

**Table 1.** Baseline Characteristics^*^.

N (%)	ESSVR (n = 324)	UC (n = 259)	Total (n = 583)
Recruitment period			
Pre-COVID < 31 Mar 2020	248 (76.5%)	197 (76.1%)	445 (76.3%)
12-m pre-COVID < 31 Mar 2019	93 (28.7%)	73 (28.2%)	166 (28.5%)
During furlough scheme *<* 30 Sep 2021	38 (11.7%)	34 (13.1%)	72 (12.3%)
Post-furlough *>* 30 Sep 2021	38 (11.7%)	28 (10.8%)	66 (11.3%)
Location of assessment			
Hospital	152 (47.6%)	121 (47.8%)	273 (47.7%)
Home	165 (51.7%)	130 (51.4%)	295 (51.6%)
Age, mean (SD)	53.7 (10.48)	54.3 (11.88)	54.0 (11.12)
Male	235 (72.8%)	165 (64.2%)	400 (69.0%)
Ethnicity			
White	254 (84.1%)	199 (83.3%)	453 (83.7%)
Black	19 (6.3%)	23 (9.6%)	42 (7.8%)
Asian	13 (4.3%)	12 (5.0%)	25 (4.6%)
Mixed	2 (0.7%)	2 (0.8%)	4 (0.7%)
Other	14 (4.6%)	3 (1.3%)	17 (3.1%)
Living with another	244 (75.5%)	203 (79.0%)	447 (77.1%)
Married/long-term relationship	212 (65.8%)	183 (71.2%)	395 (68.2%)
Carer recruited	71 (21.9%)	66 (25.5%)	137 (23.5%)
Highest qualification			
Higher education	129 (40.8%)	108 (42.9%)	237 (41.7%)
Further education	93 (29.4%)	75 (29.8%)	168 (29.6%)
Job type			
Blue collar	156 (51.5%)	120 (50.2%)	276 (50.9%)
White collar	147 (48.5%)	119 (49.8%)	266 (49.1%)
In paid/self-employment pre-stroke	301 (94.7%)	234 (94.4%)	535 (94.5%)
Pre-stroke working hours, Mean (SD)	38.3 (12.88)	37.7 (12.65)	38.1 (12.78)
Type of stroke			
Subarachnoid hemorrhage	8 (2.6%)	1 (0.4%)	9 (1.6%)
Intracerebral hemorrhage	48 (15.5%)	37 (15.6%)	85 (15.6%)
Ischemic stroke	253 (81.9%)	199 (84.0%)	452 (82.8%)
Length of hospital stay (days), Median (IQR)	4.0 (2.0, 10.0)	4.0 (2.0, 10.0)	4.0 (2.0, 10.0)
Days from stroke to randomization, Median (IQR)	28.0 (112.0, 46.0)	29.0 (13.0, 42.0)	28.0 (13.0, 44.0)
Comorbidities			
Cardiac complications	65 (20.1%)	64 (24.9%)	129 (22.2%)
Mental health problems	29 (9.0%)	26 (10.1%)	55 (9.5%)
Seizures	6 (1.9%)	6 (2.3%)	12 (2.1%)
Musculoskeletal conditions	54 (16.7%)	39 (15.2%)	93 (16.0%)
Diabetes	59 (18.3%)	40 (15.6%)	99 (17.1%)
None	165 (51.1%)	130 (50.6%)	295 (50.9%)
Post-stroke impairments			
None	161 (49.7%)	134 (51.7%)	295 (50.6%)
One	131 (40.4%)	95 (36.7%)	226 (38.8%)
Multiple	32 (9.9%)	30 (11.6%)	62 (10.6%)
Type of impairment			
Mobility^†^	119 (36.7%)	91 (35.1%)	210 (36.0%)
Aphasia^‡^	53 (16.4%)	48 (18.5%)	101 (17.3%)
Cognitive^§^	32 (9.9%)	21 (8.1%)	53 (9.1%)

* Missing: n = 11 location (other n = 4), n = 42 ethnicity, n = 3 living arrangements, n = 4 marital status, n = 15 education, n = 41 job type, n = 37 type of stroke, n = 208 length of stay, n = 3 time since stroke, n = 3 comorbidities.

† Mobility impairment = Eq-5D-5L moderate–severe problems walking about/unable to walk.

‡ Aphasia impairment = OCS picture naming task score ⩽ 3/4 (⩽ 5th centile of normative data on expressive language).

§ Cognitive impairment = OCS executive mixed task scores ⩽ 4/13 (⩽ 5th centile of normative data on Task switching/Attention).

Primary 12-month return-to-work outcome data were completed for 454/583 (77.9%) participants. Greater loss-to-follow-up occurred for secondary outcomes; 316/583 (54.2%) participants returned full 12-month questionnaires, and carer burden was available for only 54/137 (39.4%). Participants lost-to-follow-up (any timepoint) had less favorable baseline characteristics (i.e. impairments, length of hospital stay) and were more likely to have been recruited pre-COVID, female, older, non-White ethnicity, in blue-collar roles, not in paid employment, not in a relationship, living alone, and without a recruited carer. Where primary outcome data were available, participants missing secondary outcomes were less likely to have returned-to-work. Results indicated differential missing data patterns by arm (Figure S1–2). Eligibility violations (in < 1% participants), contamination (1.5%), unblinding (4.8%), withdrawals (6.0%), and deaths (< 1%) are detailed in Table S4.

The intervention commenced in 309/324 (95.4%) ESSVR participants, 244 (75.3%) were deemed to have complied,^24^ and participants attended a median 7 (IQR 4–12) sessions over 10.3 months (IQR 5.5–12.0). Median time to commence ESSVR was 9 (IQR 6–13) days post-randomization; 38 (IQR 23–56) days post-stroke. Of those commencing ESSVR, 246 (82.3%) had at least one in-person session at home, 67 (22.4%) at work, 31 (10.4%) in the community, 243 (81.3%) via telephone/video call, and 52 (17.4%) in hospital. Only 119 (40.3%) consented to OT contact with their employer, 67 (22.7%) had no employer or were self-employed and 74 (25.0%) had in-person or online employer visits. However, 60 OTs were trained and 48 delivered ESSVR for at least one participant, treating a median 6 participants (range 1–16). Analysis of ESSVR records for 39 participant–OT pairs showed OTs delivered ESSVR with acceptable overall fidelity,^21,22^ but lower fidelity to employer and family engagement.

Across methods used to capture UC,^23,25^ findings suggest there was little overall difference in health resource utilization between the ESSVR and UC groups. However, there were slightly more counselor, Speech and Language Therapy (SLT), social worker, and rehabilitation assistant appointments in the UC group, while the ESSVR group had more appointments with OTs, physiotherapist, General Practitioners (GPs), district nurses, and health care assistants. The number of secondary care outpatient visits was similar between the two groups. Inpatient stays were slightly more frequent in UC.^18^ Interview data from UC and ESSVR participants consistently identified UC provision as typically of short duration (range 2–8 weeks), predominantly focused on treating physical impairments rather than work goals. It was also perceived as poorly coordinated with limited communication between treating therapists and between therapists and participants.^19,23^

**Figure 2.**
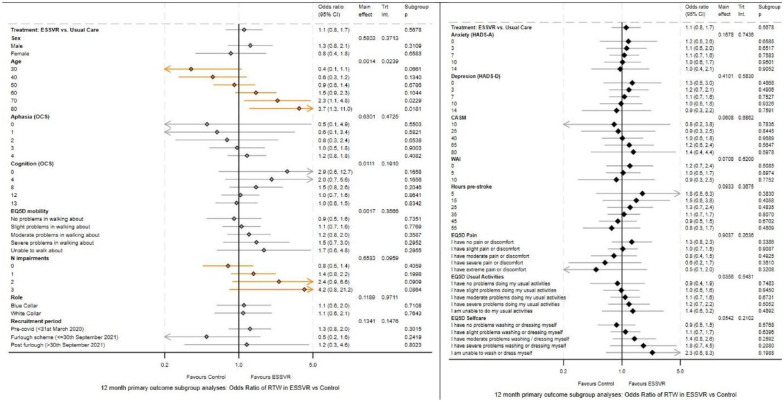
Forest plot depicting exploratory subgroup analyses.

On the 12-month primary outcome, 282/454 (62.1%) participants reported return-to-work of at least 2 h a week, 165/257 (64.2%) in ESSVR and 117/197 (59.4%) in UC, with equal proportions of participants on graded return-to-work. The adjusted OR 1.12 (95% CI 0.75–1.68, p = 0.5678) of return-to-work in ESSVR versus UC provided no evidence that ESSVR was superior to UC (Table 2). Younger participants (OR 0.97 per year, 95% CI: 0.96–0.99, p = 0.0120), those with better mobility (OR 1.43, 95% CI: 1.20–1.72, p < 0.0001) and cognition (OR 1.09, 95% CI: 1.02–1.16, p = 0.0081) were more likely to return-to-work (Table S6, Figure S4). Adjusted ORs of return-to-work in ESSVR versus UC were similar at 3 and 6 months, and there were no changes in conclusions in sensitivity analysis of complete data at 12 months (Table S5) or in analysis excluding non-compliers (135/201, 67.2% intervention compliers vs 30/56, 53.6% intervention non-compliers reported having returned-to-work). Prespecified exploratory subgroup analyses found good evidence of a differential treatment effect on the primary outcome according to participants’ age (interaction p = 0.0239). Older participants were more likely to benefit from ESSVR, and; less likely to return-to-work in UC but not ESSVR (Figure 2, Figure S4). There was some indication that participants with more post-stroke impairment were more likely to benefit from ESSVR (interaction p = 0.0959).

**Table 2.** Primary and secondary return-to-work outcomes.

	3 months			6 months			12 months		
	ESSVR (n = 324)	UC (n = 259)	Total (n = 583)	ESSVR (n = 324)	UC (n = 259)	Total (n = 583)	ESSVR (n = 324)	UC (n = 259)	Total (n = 583)
Primary outcome available	220 (67.9%)	156 (60.2%)	376 (64.5%)	227 (70.1%)	156 (60.2%)	383 (65.7%)	257 (79.3%)	197 (76.1%)	454 (77.9%)
Primary outcome: Return-to-work									
Yes	133 (60.5%)	95 (60.9%)	228 (60.6%)	152 (67.0%)	108 (69.2%)	260 (67.9%)	165 (64.2%)	117 (59.4%)	282 (62.1%)
No	87 (39.5%)	61 (39.1%)	148 (39.4%)	75 (33.0%)	48 (30.8%)	123 (32.1%)	92 (35.8%)	80 (40.6%)	172 (37.9%)
Missing	104	103	207	97	103	200	67	62	129
Odds ratio (95%CI), p-value	**1.02 (0.65, 1.60), p** **=** **0.9283**			**1.00 (0.65, 1.52), p** **=** **0.9884**			**1.12 (0.75, 1.68), p** **=** **0.5678**		
Retuned as part of:									
Graded return-to-work							35 (33.7%)	26 (34.7%)	
Supported work							2 (1.9%)	0 (0.0%)	
None							28 (26.9%)	31 (41.3%)	
Other							39(37.5%)	18(24.0%)	
Missing							61	42	
Secondary outcomes:	In those reporting return to work at follow-up								
Stroke impacted work status^*^	103/113 (91.2%)	73/85 (85.9%)	176/198 (88.9%)	78/127 (61.4%)	54/89 (60.7%)	132/216 (61.1%)	51/105 (48.6%)	34/77 (44.2%)	85/182 (46.7%)
Hours									
Change in working hours	66/108 (61.1%)	39/80 (48.8%)	105/188 (55.9%)	59/124 (47.6%)	33/87 (37.9%)	92/211 (43.6%)	41/103 (39.8%)	24/75 (32.0%)	65/178 (36.5%)
If yes, current working hours, M (SD)	18.3 (12.24), n = 51	20.3 (12.15), n = 35	19.1 (12.17), n = 86	19.9 (11.11), n = 31	24.2 (8.90), n = 18	21.5 (10.47), n = 49	28.4 (11.65), n = 33	31.5 (11.71), n = 15	29.4 (11.64), n = 48
Pre-stroke working hours, M (SD)	41.2 (12.04), n = 118	37.3 (12.89), n = 78	39.7 (12.50), n = 196	38.7 (12.45), n = 135	38.5 (12.89), n = 94	38.6 (12.61), n = 229	39.0 (11.77), n = 145	39.3 (10.78), n = 103	39.1 (11.35), n = 248
Days worked									
Have had to take time off	91/111 (82.0%)	61/83 (73.5%)	152/194 (78.4%)	42/124 (34.4%)	31/85 (36.5%)	73/207 (35.3%)	22/98 (22.4%)	14/72 (19.4%)	36/170 (21.2%)
If yes, weeks taken off, M (SD)	10.2 (4.30), n = 78	10.3 (5.97), n = 54	10.2 (5.02), n = 132	6.7 (5.91), n = 32	5.9 (5.04), n = 23	6.3 (5.52), n = 55	13.5 (15.78), n = 15	7.8 (8.26), n = 11	11.1 (13.22), n = 26
Role									
Changed role	12/102 (11.8%)	9/75 (12.0%)	21/177 (11.9%)	12/122 (9.8%)	15/87 (17.2%)	27/209 (12.9%)	13/103 (12.6%)	9/76 (11.8%)	22/179 (12.3%)

Bold text related to estimated adjusted treatment effects.

* Over the past 3 months.

In participants who had returned-to-work at 12 months (Table 2), 41/103 (39.8%) ESSVR versus 24/75 (32.0%) UC participants reported a change in working hours, of whom the mean weekly hours were reduced in ESSVR (28.4, SD 11.65) compared to UC (31.5, SD 11.71). A similar pattern was observed at 3 and 6 months but with a decreasing proportion of participants with changes in working hours and increased working hours over time. At 12 months, more ESSVR participants (22/98, 22.4%) reported having taken time off due to their stroke over the past 3 months compared to UC (14/72, 19.4%), and 13/103 (12.6%) ESSVR versus 9/76 (11.8%) UC participants reported a change in role.

Other secondary outcomes (Table 3, Figure S3) were largely similar, with small differences between trial arms and provided no evidence that ESSVR was superior to UC. However, participants tended to have slightly improved outcomes in UC compared to ESSVR, and UC participants reported statistically significantly better functional ability (NEADL: MD −3.37, 95% CI −6.26 to −0.48, p = 0.0230) and carer burden (MSCI: MD 2.52, 95% CI 0.63 to 4.41, p = 0.0095) at 12 months in multiply imputed analyses. Statistically significant effects were not observed at other timepoints, or in sensitivity analysis (Table S4) and should be interpreted with caution given substantial loss-to-follow-up. For further exploratory comparison of secondary outcomes, see Table S9.

**Table 3.** Secondary Outcomes^†^.

	Baseline			3 months			6 months			12 months		
	ESSVR (n = 324)	UC (n = 259)	Total (n = 583)	ESSVR (n = 324)	UC (n = 259)	MD (95%CI), p-value	ESSVR (n = 324)	UC (n = 259)	MD (95%CI), p-value	ESSVR (n = 324)	UC (n = 259)	MD (95%CI), p-value
Questionnaire returned				195 (60.2%)	144 (55.6%)	339 (58.1%)	199 (61.4%)	142 (54.8%)	341 (58.5%)	182 (56.2%)	134 (51.7%)	316 (54.2%)
Mood: HADs-Anxiety^‡^, M (SD)	6.6 (4.38), n = 314	7.0 (4.65), n = 247	6.8 (4.50), n = 561	7.5 (4.86), n = 179	7.4 (4.45), n = 127	**0.43 (−0.48, 1.34), p** **=** **0.3518**	6.5 (4.74), n = 180	6.7 (4.44), n = 127	**0.60 (−0.32, 1.53), p** **=** **0.2000**	6.8 (5.01), n = 155	7.2 (4.56), n = 104	**0.24 (−0.71, 1.20),** **p** **=** **0.6174**
Normal (0–7)	187 (59.6%)	134 (54.3%)	321 (57.2%)	96 (53.6%)	64 (50.4%)	160 (52.3%)	109 (60.6%)	76 (59.8%)	185 (60.3%)	92 (59.4%)	62 (59.6%)	154 (59.5%)
Mild (8–10)	67 (21.3%)	56 (22.7%)	123 (21.9%)	36 (20.1%)	32 (25.2%)	68 (22.2%)	33 (18.3%)	23 (18.1%)	56 (18.2%)	25 (16.1%)	15 (14.4%)	40 (15.4%)
Moderate (11–14)	45 (14.3%)	39 (15.8%)	84 (15.0%)	31 (17.3%)	24 (18.9%)	55 (18.0%)	25 (13.9%)	21 (16.5%)	46 (15.0%)	24 (15.5%)	21 (20.2%)	45 (17.4%)
Severe (15–21)	15(4.8%)	18(7.3%)	33(5.9%)	16(8.9%)	7(5.5%)	23(7.5%)	13(7.2%)	7(5.5%)	20(6.5%)	14(9.0%)	6(5.8%)	20(7.7%)
Mood: HADs-Depression^‡^, M (SD)	6.1 (3.94), n = 311	6.2 (4.18), n = 247	6.1 (4.04), n = 558	6.3 (4.38), n = 179	5.9 (3.98), n = 127	**0.40 (−0.49, 1.29),** **p** **=** **0.3772**	5.9 (4.28), n = 180	5.6 (4.14), n = 128	**0.56 (−0.36, 1.48),** **p** **=** **0.2305**	5.7 (4.59), n = 158	5.4 (4.13), n = 105	**0.58 (−0.40, 1.56),** **p** **=** **0.2416**
Normal (0–7)	201 (64.6%)	156 (63.2%)	357 (64.0%)	108 (60.3%)	86 (67.7%)	194 (63.4%)	119 (66.1%)	90 (70.3%)	209 (67.9%)	114 (72.2%)	78 (74.3%)	192 (73.0%)
Mild (8–10)	68 (21.9%)	50 (20.2%)	118 (21.1%)	40 (22.3%)	21 (16.5%)	61 (19.9%)	35 (19.4%)	18 (14.1%)	53 (17.2%)	19 (12.0%)	15 (14.3%)	34 (12.9%)
Moderate (11–14)	32(10.3%)	31(12.6%)	63(11.3%)	20(11.2%)	18(14.2%)	38(12.4%)	17(9.4%)	17(13.3%)	34(11.0%)	16(10.1%)	9(8.6%)	25(9.5%)
Severe (15–21)	10 (3.2%)	10 (4.0%)	20 (3.6%)	11 (6.1%)	2 (1.6%)	13 (4.2%)	9 (5.0%)	3 (2.3%)	12 (3.9%)	9 (5.7%)	3 (2.9%)	12 (4.6%)
Functional ability: NEADL, M (SD)	61.4 (12.21) ,n = 315	62.5 (11.04), n = 252	61.9 (11.71), n = 567				54.9 (13.08), n = 179	56.3 (11.92), n = 129	**−1.05 (−3.96, 1.86),** **p** **=** **0.4755**	54.3 (13.20), n = 157	57.9 (10.75), n = 109	**−3.37(−6.26, −0.48),** **p** **=** 0.0230**
Participation: CIQ-R Social Integration, M (SD)	7.1 (1.89), n = 315	7.1 (1.92), n = 250	7.1 (1.90), n = 565							6.0 (2.24), n = 153	6.5 (2.16), n = 109	**−0.36 (−0.86, 0.13),** **p** **=** **0.1493**
Participation: CIQ-R Productivity, M (SD)	5.6 (1.18), n = 285	5.6 (1.22), n = 234	5.6 (1.20), n = 519							4.3 (2.04), n = 149	4.6 (2.03), n = 106	**−0.40(−0.82, 0.01),** **p** **=** **0.0571**
Work self-efficacy: WAI, M (SD)	3.7 (3.00), n = 311	3.6 (3.07), n = 246	3.6 (3.03), n = 557	5.0 (3.14), n = 182	5.4 (3.13), n = 127	**−0.44 (−1.06, 0.17), p** **=** **0.1551**	6.0 (2.71), n = 180	6.2 (3.07), n = 129	**−0.27 (−0.84, 0.30), p** **=** **0.3537**	6.2 (3.08), n = 154	6.6 (2.82), n = 111	**−0.45 (−1.18, 0.28),** **p** **=** **0.2226**
Post-stroke confidence: CASM, M (SD)	51.0 (13.09), n = 312	50.9 (12.83), n = 236	50.9 (12.97), n = 548							51.2 (15.42), n = 149	52.0 (13.89), n = 104	**−0.79 (−3.64, 2.06)** **, p** **=** **0.5837**
Carer burden: MSCI^‡^, M (SD)	9.0 (6.08), n = 67	8.5 (6.23), n = 61	8.7 (6.13), n = 128	8.3 (6.47), n = 37	7.7 (6.01), n = 24	**−0.27 (−2.08, 1.54), p** **=** **0.7681**	7.5 (6.68), n = 38	6.2 (5.37), n = 18	**0.87 (−1.59, 3.32), p** **=** **0.4858**	8.1 (6.08), n = 37	3.9 (4.31), n = 17	**2.52 (0.63, 4.41),** **p** **=** 0.0095**

† Bold text related to estimated treatment effects. MD (95% CI) represents the adjusted mean difference between treatment groups, ESSVR/UC. HADS scores range 0–21, higher scores indicate more severe anxiety/depression. NEADL scores range 0–66, higher scores indicate greater functional ability. CIQ-R Social Integration scores range 0–10, productivity scores 0–7; higher scores indicate greater community integration. WAI scores range 0–10, higher values indicate better work ability. CASM Scores range 0–81, higher scores indicate greater confidence. MCSI scores range 0–26, higher scores indicate greater carer burden. ** indicates statistically significant effects.

‡ Lower scores indicate better outcomes for measures with a ^‡^, otherwise higher scores indicate better outcomes.

There were no Related and Unexpected Serious Adverse Events. Self-reported safety outcomes were similar for both groups (Table S10).

## Discussion

### Main findings

In stroke survivors working at stroke onset, we found no quantitative evidence of benefit of ESSVR over UC in self-reported return-to-work, mood, functional ability, social participation, work self-efficacy, post-stroke confidence, or carer burden. These findings are in a predominantly male (69%, consistent with UK stroke registry data),^4^ relatively young (mean 54 years) and mild-to-moderate sample of stroke survivors. The study was conducted during a pandemic, a period marked by significant changes in UK work practices (see supplementary material for further reflection) and results are influenced by high levels of missing data for secondary outcomes and some limitations in employer engagement.

Although 5% more ESSVR than UC participants returned-to-work (64.2% vs 59.4%), this was not statistically significant. More UC participants returned-to-work than expected, more than double that observed in our feasibility trial (26%). Possibly due to case-mix, pandemic effects, and recent evidence suggesting higher rates, in younger stroke survivors, motivated to return-to-work.^38^

Only 11% of RETAKE participants had more than one impairment in mobility, cognition, or expressive language indicative of a mild–moderate severity sample. Participants were also predominantly male, White, well educated, and half were employed in white-collar roles. All were significant predictors of return-to-work.^38^ These stroke survivors may be capable of self-advocating and navigating return-to-work without intensive ESSVR support.

Exploratory subgroup analyses found ESSVR was more likely to benefit people disadvantaged by age and impairment. However, further research is required to confirm these findings.

In participants who returned-to-work, more ESSVR participants reported changes in working hours and taking time off compared to UC, suggesting ESSVR might influence return to modified work, possibly enabling those who might not otherwise return-to-work to do so, or ensuring work is sustainable and work-life balanced maintained.

Our finding of slightly improved outcomes in UC compared to ESSVR on secondary outcomes, particularly 12-month functional ability and carer burden, should be interpreted with caution. Improvements largely represented very small effect sizes < 0.2^39^ and were unreliable due to high levels of missing data.

### Strengths

Despite challenges recruiting to multicentre stroke trials^40^ and a global pandemic, this first, large, powered, UK trial of ESSVR achieved our revised target, and almost 80% follow-up of primary 12-month return-to-work outcomes.

Inclusion criteria were broad, aiming to support return-to paid or unpaid work irrespective of age recognizing increases in state pension age, the value of work to health and its meaning in people’s lives.^6^

ESSVR was co-developed with expert service users and providers following MRC guidance,^27^ drawing on best available evidence and clinical guidelines at the time.^41,42^ It was valued by participants, OTs, and employers,^30^ and compliance was good and fidelity acceptable.^22^

Our seven PPI representatives met 6-monthly to define our primary outcome, inform research design, OT training, participant resources, troubleshoot issues, interpretation, and dissemination.^43^

### Limitations

The pandemic changed the health care and employment contexts in which ESSVR was delivered. It also changed the meaning of work in people’s lives and influenced the “great retirement”^44^ (Further details see supplementary material). It impacted RETAKE recruitment, intervention delivery, data collection, and follow-up. RETAKE paused to recruitment 1 week after the first UK COVID-19 lockdown was mandated with the trial completed in just 28.5% participants. Most post-COVID intervention delivery occurred online or by phone, rather than face-to-face as in the feasibility trial, with more time spent addressing current issues, and offering psychological support and increased difficulty engaging employers.^24^ This was possibly in response to disruption caused to people’s lives,^45^ heightened anxiety,^46,47^ limited access to NHS services,^48^ and COVID-19 symptoms, such as fatigue, possibly compounding that related to stroke.^2,49^ During the pandemic widespread implementation of telehealth across the NHS, changed rehabilitation delivery, raising concerns about digital exclusion.^50^ It is possible that telehealth enabled UC further advantaged socially advantaged people with fewer disabilities. The impact of COVID-19 infection on work ability^51^ led to an NHS England-led nationwide initiative^52^ to develop resources for NHS health care professionals to support return-to-work following COVID-19 infection. This possibly equipped OTs with VR skills that were transferable to stroke.

The pandemic also impacted the employment context. Efforts to minimize COVID-19 spread^37^ necessitated flexible home-based working and widespread implementation of videoconferencing software possibly advantaging the least disabled, and people conversant in and with access to technology. Efforts to facilitate remote working and support employees during lockdowns, coupled with heightened awareness of pandemic-related health inequity^53^ and labor shortages,^54^ may have expedited employer awareness of Equality, Diversity, and Inclusion. These changes compromised core intervention mechanisms (employer engagement and education, cross-boundary working, negotiating reasonable adjustments). The pandemic increased the length of the trial to over 5 years. In this time, new guidelines^10,11,52^ advocating the need for VR, highlighted the need for “early intervention,” and the Stroke Sentinel National Audit Program, introduced VR-specific questions to its audit, influencing changes in clinical practice.^55^ Despite providing training and support to recruiting clinical research network staff, only 10% of participants were cognitively impaired and 17% had aphasia. High staff turnover,^56^ and use of pre-recorded training resources following the pandemic, may have contributed. Interviews with recruiting teams highlighted varied perceptions regarding the appropriateness of recruiting patients “early after stroke.”

Despite efforts to maintain participant engagement, full questionnaire completion was low with secondary outcomes missing for more than half the sample. Those lost to follow-up tended to represent more severe stroke, with differential missing data patterns by arm, limiting the reliability of comparison between groups on secondary outcomes. Reducing questionnaire length or collecting data via other means (i.e. medical records) may have improved completion rates. Contractual issues meant it was not possible to obtain aggregated non-identifiable data on work status via the DWP.

We were unable to explore the effect of contract type or flexible working in relation to outcomes, and recommend future data collection to include employment on zero hours contracts and ability to work remotely. The NIH Stroke Scale for quantifying stroke severity was not collected; therefore, we quantified using the number of impairments in mobility, aphasia, and cognition.

### Future research directions

Younger age, high education, believing work is important and self-expectations of return-to-work are positive predictors for return to work^57,58^ (refs). These factors have undoubtedly influenced the findings of this trial, which recruited a predominantly male, relatively young (mean 54 years) and mild-to-moderate sample of stroke survivors and where intention to return-to-work was a trial inclusion criterion. Where resources are limited, our findings suggest ESSVR should be targeted, potentially at older patients and those with greater post-stroke impairment. Further research to confirm this finding is needed, as is research to better understand the needs of people with aphasia, less well-educated stroke survivors on lower incomes and younger stroke survivors with little or no residual disability who are able to self-advocate and motivated to return.

Longer follow-up studies are needed. Future trials should consider minimizing data collection to reduce participant burden, and resourcing data collection support for those who need it; stratify by stroke severity; and comprehensively document UC. Involving PPI members in training recruiters may also help overcome recruitment bias.

## Conclusion

The quantitative findings from this first definitive RCT of a stroke specialist VR intervention found no evidence of benefit of ESSVR on return-to-work. The pandemic changed the world of work irreversibly, and health care delivery beyond anything that could have been anticipated in the trial lifetime. It changed the meaning of work in people’s lives, increasing rates of early retirement, and compromised key ESSVR mechanisms, the overall effectiveness of the intervention, our primary outcome, and trial delivery.

## Supplemental Material

sj-docx-1-wso-10.1177_17474930241306693 – Supplemental material for Effectiveness of early vocational rehabilitation versus usual care to support RETurn to work after stroKE: A pragmatic, parallel-arm multicenter, randomized controlled trial Supplemental material, sj-docx-1-wso-10.1177_17474930241306693 for Effectiveness of early vocational rehabilitation versus usual care to support RETurn to work after stroKE: A pragmatic, parallel-arm multicenter, randomized controlled trial by KA Radford, A Wright-Hughes, E Thompson, DJ Clarke, J Phillips, J Holmes, K Powers, D Trusson, K Craven, C Watkins, A Bowen, C McKevitt, J Stevens, JD Murray, RJ O’Connor, S Pyne, H Risebro, R Cameron, TH Sach, F Day and AJ Farrin in International Journal of Stroke
