# Supplementary material for: Effectiveness of early vocational rehabilitation versus usual care to support RETurn to work after stroKE: A pragmatic, parallel-arm multicenter, randomized controlled trial
Source: Int J Stroke. 2024 Dec 31;20(4):471–85. doi: 10.1177/17474930241306693 (PMC11951359; doi:10.1177/17474930241306693)
Supplement: sj-docx-1-wso-10.1177_17474930241306693 – Supplemental material for Effectiveness of early vocational rehabilitation versus usual care to support RETurn to work after stroKE: A pragmatic, parallel-arm multicenter, randomized controlled trial [file sj-docx-1-wso-10.1177_17474930241306693.docx]

**SUPPLEMENTARY MATERIAL**

# **ADDITIONAL ANALYSIS METHODS**

**Partially nested regression**

Analyses allowed for clustering of participants according to the ‘main’ OT who delivered ESSVR in the intervention arm by including OT as a random effect. Participants randomised to the intervention arm who did not commence the intervention, and participants in the control arm, were treated as clusters of size one. The mixed model applied a Satterthwaite correction and where possible allowed for heteroscedasticity (i.e. a different variance) to distinguish between clusters that were defined by an OT in the intervention arm and individual clusters in the control arm.

**Multiple Imputation**

Baseline variables were included in the imputation models where they were found to be predictive of missingness, with relation to (i) the return-to-work outcome or of full questionnaire completion, (ii) any follow-up time point (3, 6 or 12 months), and (iii) overall or differentially by arm. Variables predictive of missingness and thus included in the imputation models were: recruitment period, carer recruitment, marital status, living arrangements, ethnicity, stroke and job type, number of post stroke impairments, baseline assessment location and questionnaire scores (NEADL, HADS-A, HADS-D, CASM, CIQ total).

Participants lost-to-follow-up (at any timepoint) had less favourable baseline characteristics on impairments, length of hospital stay, stroke type, confidence, anxiety, depression, functional ability; and were more likely to have been recruited pre-covid in hospital, female, older, non-white ethnicity, in blue-collar roles, not in paid employment, not in a relationship, living alone, and without a recruited carer. Where primary outcome data were available, participants missing secondary outcomes were also less likely to have returned-to-work. Results also indicated differential missing data patterns by arm on impairments, community integration, length of hospital stay, recruitment pre-covid, age, gender, marital status, and work status (Figure-A1).

# **ADDITIONAL COVID-19 INFORMATION**

**Methods**

Between September 2020 and July 2021, researchers collected information on the impact of COVID-19 on participants during follow-up phone calls.

**Results**

Characteristics of participants according to carer recruitment and the pandemic were relatively similar (Table-S2).

At 12-months, 21/47 (44.7%) participants in work prior to COVID-19 reported their work status changed due to COVID-19 with just over half not returned to work. Two thirds (42/63, 66.7%) were social distancing, a quarter (16/63, 25.4%) were living life as normal, and the remainder self-isolating. Just under a quarter had applied for or received financial help, the majority via the UK Coronavirus Job Retention (furlough) scheme (Table-S7-8).

**Discussion**

The pandemic was the greatest limitation. It changed the healthcare and employment contexts in which ESSVR was delivered, the meaning of work in people’s lives and influenced the ‘great retirement’(1). It emerged at a critical point in RETAKE, impacting recruitment, intervention delivery, data collection and follow up. RETAKE paused to recruitment one week after the first UK COVID-19 lockdown was mandated and the furlough scheme introduced (supporting employers to retain and pay staff while businesses were closed), having recruited 76.3% of our final sample across 21 sites but with trial completed in 28.5% participants.

Government directives to ‘protect the NHS’ led to redeployment of several of the 50 trained RETAKE OTs to COVID-19 activity. Routine procedures and non-Covid research were de-prioritised. Some stroke rehabilitation services were reconfigured. Following initial lockdown, five sites closed; 20 OTs were unable to continue.

Whilst the trial was intended for face-to-face delivery, we rapidly re-designed trial processes and training resources to support remote recruitment and delivery of ESSVR. Fewer intervention sessions were delivered in-person post-Covid whilst delivery online or by phone increased substantially and there was increased difficulty engaging employers - a core underlying mechanism of ESSVR(2). The focus of ESSVR also changed. Most time was spent addressing current issues, ~50% of sessions(2) versus 16% in the feasibility trial, with more time spent on fatigue management and informal psychological support. This was possibly in response to disruption caused to people’s lives(3), heightened anxiety(4, 5), limited access to NHS services(6) and Covid-19 symptoms, such as fatigue, possibly compounding that related to stroke(7, 8).

ESSVR is a complex intervention and dependent on the delivery context(9). It relies on VR-trained OTs crossing service boundaries within and between health and the employment sector however the pandemic changed the employment context. The impact of Covid-19 infection on ability to work(10) led to an NHS England-led nationwide initiative(11) to develop resources for NHS healthcare professionals to support return-to-work following Covid-19 infection. This possibly equipped OTs with VR skills that were transferable to stroke. Widespread implementation of telehealth across the NHS, changed rehabilitation delivery, raising concerns about digital exclusion(12). UC became more accessible for people with fewer disabilities, and those conversant in and with access to technologies.

Efforts to minimise COVID-19 spread and lockdown measures(13) also necessitated home-based working and led to widespread implementation of videoconferencing software and flexible working practices. Efforts to facilitate remote working and support employees during lockdowns, coupled with heightened awareness of pandemic-related health inequity(14) and labour shortages[55], may have expedited employer awareness of Equality, Diversity and Inclusion. These changes compromised core intervention mechanisms (employer engagement and education, cross-boundary working, negotiating reasonable adjustments) and impacted on usual care. Home-based working also mitigated against the disabling effects of neurological fatigue exacerbated by travelling to work and reduced the length of the working day(3). People with other long-term health conditions such as MS, and inflammatory arthritis reported increased productivity(15, 16).

The pandemic increased the length of the trial to over five years. In this time new guidelines(11, 17, 18) advocating the need for VR, highlighted the need for ‘early intervention’, and the Stroke Sentinel National Audit Programme, introduced VR specific questions to its audit, influencing changes in clinical practice and usual care(19).

# **ADDITIONAL TABLES AND FIGURES**

**Table-S1 Demographics of screened population, eligible or discharged population and randomised population**

|  | **Screened Population (n=3672)** | **Eligible or identified to approach post discharge (n=1918)** | **Introduced to Study (n=1316)** | **Randomised Population (n=583)** |
| --- | --- | --- | --- | --- |
| **Age** |  |  |  |  |
| Mean (SD) | 64.2 (15.77) | 55.8 (11.70) | 54.9 (11.24) | 54.0 (11.12) |
| Median (range) | 63.0 (17, 106) | 56.0 (18, 93) | 56.0 (19, 93) | 55.0 (19, 93) |
| Missing | 39 | 31 | 30 | 0 |
| N | 3633 | 1887 | 1286 | 583 |
| **Gender** |  |  |  |  |
| Male | 2268 (62.1%) | 1333 (69.9%) | 925 (70.8%) | 400 (69.0%) |
| Female | 1384 (37.9%) | 574 (30.1%) | 382 (29.2%) | 180 (31.0%) |
| Missing | 20 | 11 | 9 | 3 |
| **Ethnicity** |  |  |  |  |
| White | 2768 (80.8%) | 1331 (74.0%) | 904 (73.7%) | 453 (80.3%) |
| Black | 181 (5.3%) | 137 (7.6%) | 89 (7.3%) | 42 (7.4%) |
| Asian | 200 (5.8%) | 129 (7.2%) | 102 (8.3%) | 25 (4.4%) |
| Mixed | 13 (0.4%) | 10 (0.6%) | 6 (0.5%) | 4 (0.7%) |
| Other ethnic group | 77 (2.2%) | 56 (3.1%) | 40 (3.3%) | 17 (3.0%) |
| Not stated | 185 (5.4%) | 136 (7.6%) | 86 (7.0%) | 23 (4.1%) |
| Missing | 248 | 119 | 89 | 19 |

|  | **Total**  **(n=583)** | **Population with a recruited carer**  **(n=137)** | **Recruitment period** | | |
| --- | --- | --- | --- | --- | --- |
|  |  |  | **Pre-covid**  **(<31.03.20)**  **(n=445)** | **Furlough scheme (<=30.09.21)**  **(n=72)** | **Post furlough**  **(>30.09.2021)**  **(n=66)** |
| **Recruitment period, n (%)** |  |  |  |  |  |
| Pre-covid *<31.03.20* | 445 (76.3%) | 114 (83.2%) |  |  |  |
| During furlough scheme *<30.09.21* | 72 (12.3%) | 16 (11.7%) |  |  |  |
| Post furlough *>30.09.21* | 66 (11.3%) | 7 (5.1%) |  |  |  |
| **Location of assessment, n (%)** |  |  |  |  |  |
| Hospital | 273 (47.7%) | 42 (31.3%) | 200 (46.0%) | 37 (51.4%) | 36 (55.4%) |
| Home | 295 (51.6%) | 92 (68.7%) | 235 (54.0%) | 35 (48.6%) | 25 (38.5%) |
| **Age, mean (SD)** | 54.0 (11.12) | 54.2 (11.86) | 54.0 (11.02) | 53.9 (11.38) | 53.8 (11.66) |
| **Male, n (%)** | 400 (69.0%) | 97 (70.8%) | 309 (69.4%) | 51 (70.8%) | 40 (63.5%) |
| **Ethnicity, n (%)** |  |  |  |  |  |
| White | 453 (83.7%) | 105 (87.5%) | 353 (84.7%) | 49 (79.0%) | 51 (82.3%) |
| Black | 42 (7.8%) | 7 (5.8%) | 31 (7.4%) | 6 (9.7%) | 5 (8.1%) |
| Asian | 25 (4.6%) | 4 (3.3%) | 16 (3.8%) | 4 (6.5%) | 5 (8.1%) |
| Mixed | 4 (0.7%) | 1 (0.8%) | 3 (0.7%) | 0 | 1 (1.6%) |
| Other ethnic group | 17 (3.1%) | 3 (2.5%) | 14 (3.4%) | 3 (4.8%) | 0 |
| **Living with another person, n (%)** | 447 (77.1%) | 123 (89.8%) | 341 (76.6%) | 55 (76.4%) | 51 (81.0%) |
| **Married, living with or in a long-term relationship, n (%)** | 395 (68.2%) | 118 (86.1%) | 299 (67.3%) | 48 (66.7%) | 48 (76.2%) |
| **Carer recruited, n(%)** | 137 (23.5%) | 137 (100.0%) | 114 (25.6%) | 16 (22.2%) | 7 (10.6%) |
| **Highest education, n(%)** |  |  |  |  |  |
| Higher education qualification | 237 (41.7%) | 56 (42.1%) | 166 (38.2%) | 39 (54.9%) | 32 (50.8%) |
| Further education qualification | 168 (29.6%) | 38 (28.6%) | 139 (32.0%) | 12 (16.9%) | 17 (27.0%) |
| **Job Collar Type, n(%)** |  |  |  |  |  |
| Blue Collar | 276 (50.9%) | 64 (51.2%) | 209 (50.7%) | 32 (47.8%) | 35 (55.6%) |
| White Collar | 266 (49.1%) | 61 (48.8%) | 203 (49.3%) | 35 (52.2%) | 28 (44.4%) |
| **In paid employment/Self-employed pre-stroke, n(%)** | 535 (94.5%) | 122 (93.8%) | 407 (94.4%) | 66 (94.3%) | 62 (95.4%) |
|  |  |  |  |  |  |
| **Type of stroke, n (%)** |  |  |  |  |  |
| Subarachnoid haemorrhage | 9 (1.6%) | 2 (1.6%) | 8 (1.9%) | 0 | 1 (1.6%) |
| Intracerebral haemorrhage | 85 (15.6%) | 20 (15.7%) | 62 (15.0%) | 10 (13.9%) | 13 (21.0%) |
| Ischaemic stroke | 452 (82.8%) | 105 (82.7%) | 342 (83.0%) | 62 (86.1%) | 48 (77.4%) |
| **Length of hospital stay (days), Median (IQR)** | 4.0 (2.0, 10.0) | 4.0 (2.0, 10.0) | 4.0 (2.0, 10.0) | 3.0 (1.0, 7.0) | 4.0 (2.0, 9.0) |
| **Time from stroke to randomisation (days), Median (IQR)** | 28.0 (13.0, 44.0) | 30.5 (18.56) | 28.0 (13.0, 43.0) | 22.0 (10.5, 39.0) | 37.0 (11.0, 58.0) |
| **Comorbidities, n (%)** |  |  |  |  |  |
| Cardiac Complications | 129 (22.2%) | 36 (26.3%) | 109 (24.5%) | 12 (16.7%) | 8 (12.7%) |
| Mental health Problems | 55 (9.5%) | 12 (8.8%) | 44 (9.9%) | 6 (8.3%) | 5 (7.9%) |
| Seizures | 12 (2.1%) | 2 (1.5%) | 11 (2.5%) | 1 (1.4%) | 0 |
| Musculoskeletal Conditions | 93 (16.0%) | 20 (14.6%) | 78 (17.5%) | 7 (9.7%) | 8 (12.7%) |
| Diabetes | 99 (17.1%) | 30 (21.9%) | 76 (17.1%) | 12 (16.7%) | 11 (17.5%) |
| None | 295 (50.9%) | 75 (54.7%) | 217 (48.8%) | 43 (59.7%) | 35 (55.6%) |
| **N Post stroke impairments, n (%)** |  |  |  |  |  |
| None | 295 (50.6%) | 75 (54.7%) | 227 (51.0%) | 36 (50.0%) | 32 (48.5%) |
| One | 226 (38.8%) | 52 (38.0%) | 173 (38.9%) | 28 (38.9%) | 25 (37.9%) |
| Multiple | 62 (10.6%) | 10 (7.3%) | 45 (10.1%) | 8 (11.1%) | 9 (13.6%) |
| **Type of post stroke impairment, n (%)** |  |  |  |  |  |
| Mobility (EQ-5D-5L Mobility Score^[[1]](#footnote-1)^) | 210 (36.0%) | 40 (29.2%) | 154 (34.6%) | 27 (37.5%) | 29 (43.9%) |
| Aphasia (OCS Picture Naming Score^[[2]](#footnote-2)^) | 101 (17.3%) | 24 (17.5%) | 85 (19.1%) | 8 (11.1%) | 8 (12.1%) |
| Cognitive (OCS Mixed Score^[[3]](#footnote-3)^) | 53 (9.1%) | 9 (6.6%) | 34 (7.6%) | 11 (15.3%) | 8 (12.1%) |

**Table-S2 Baseline Characteristics by carer and recruitment period populations**

**Table-S3 Full baseline Oxford Cognitive Screen^[[4]](#footnote-4)^**

|  | **ESSVR (n=324)** | **UC (n=259)** | **Total (n=583)** |
| --- | --- | --- | --- |
| **OCS version** |  |  |  |
| Version A | 288/322 (89.4%) | 224/257 (87.2%) | 512/579 (88.4%) |
| Version B | 34/322 (10.6%) | 33/257 (12.8%) | 67/579 (11.6%) |
| **MEMORY** |  |  |  |
| **Orientation** |  |  |  |
| Mean (SD) | 3.9 (0.28) | 4.0 (0.25) | 3.9 (0.26) |
| Impairment (score<4), n/N (%) | 15/320 (4.7%) | 10/257 (3.9%) | 25/577 (4.3%) |
| **Verbal memory: Recognition** |  |  |  |
| Mean (SD) | 3.6 (0.81) | 3.5 (0.98) | 3.6 (0.89) |
| Impairment (score<3), n/N (%) | 32/317 (10.1%) | 35/257 (13.6%) | 67/574 (11.7%) |
| **Episodic memory: Recognition** |  |  |  |
| Mean (SD) | 3.8 (0.42) | 3.8 (0.50) | 3.8 (0.46) |
| Impairment (score<3), n/N (%) | 5/320 (1.6%) | 10/255 (3.9%) | 15/575 (2.6%) |
| **VISUAL FIELD ASSESSMENT** |  |  |  |
| Mean (SD) | 3.9 (0.55) | 3.9 (0.45) | 3.9 (0.50) |
| Impairment (score<4), n/N(%) | 24/302 (8.4%) | 21/249 (8.4%) | 45/551 (8.2%) |
| **LANGUAGE** |  |  |  |
| **Picture Naming Score** |  |  |  |
| Mean (SD) | 3.7 (0.73) | 3.7 (0.71) | 3.7 (0.72) |
| Impairment (score <=3), n/N (%) | 53/324 (16.4%) | 48/259 (18.5%) | 101/583 (17.3%) |
| **Semantics / Picture Pointing** |  |  |  |
| Mean (SD) | 3.0 (0.20) | 3.0 (0.15) | 3.0 (0.18) |
| Impairment (score<3), n/N (%) | 5/320 (1.6%) | 3/257 (1.2%) | 8/577 (1.4%) |
| **Sentence reading** |  |  |  |
| Mean (SD) | 13.9 (3.02) | 13.8 (2.99) | 13.8 (3.00) |
| Impairment (score<14), n/N (%) | 52/317 (16.4%) | 53/257 (20.6%) | 105/574 (18.3%) |
| **NUMERICAL COGNITION** |  |  |  |
| **Number writing** |  |  |  |
| Mean (SD) | 2.8 (0.62) | 2.7 (0.69) | 2.8 (0.65) |
| Impairment (score<3), n/N (%) | 37/317 (11.7%) | 38/257 (14.8%) | 75/574 (13.1%) |
| **Calculations** |  |  |  |
| Mean (SD) | 3.7 (0.58) | 3.6 (0.67) | 3.7 (0.62) |
| Impairment (score<3), n/N (%) | 14/319 (4.4%) | 21/257 (8.2%) | 35/576 (6.1%) |
| **PRAXIS (Imitation)** |  |  |  |
| Mean (SD) | 10.8 (2.23) | 10.8 (2.17) | 10.8 (2.20) |
| Impairment (score<8), n/N (%) | 28/320 (8.8%) | 18/256 (7.0%) | 46/576 (8.0%) |
| **ATTENTION** |  |  |  |
| **Broken hearts - overall accuracy** |  |  |  |
| Mean (SD) | 44.6 (8.76) | 44.5 (8.32) | 44.5 (8.56) |
| Impairment (score<42), n/N (%) | 52/317 (16.4%) | 50/252 (19.8%) | 102/569 (17.9%) |
| **Broken hearts - space asymmetry** |  |  |  |
| Mean (SD) | 0.8 (5.47) | 0.6 (5.19) | 0.7 (5.35) |
| Impairment (score>\|2\|), n/N (%) | 68/300 (22.7%) | 57/241 (23.7%) | 125/541 (23.1%) |
| **Broken hearts - object asymmetry** |  |  |  |
| Mean (SD) | 0.0 (1.38) | -0.1 (2.45) | -0.0 (1.93) |
| Impairment (score not 0), n/N (%) | 66/300 (22.0%) | 60/243 (24.7%) | 126/543 (23.2%) |
| **Executive Mixed Score - cognition** |  |  |  |
| Mean (SD) | 11.1 (3.64) | 11.0 (3.52) | 11.0 (3.59) |
| Impairment (score <=4), n/N (%) | 32/324 (9.9%) | 21/259 (8.1%) | 53/583 (9.1%) |
| **Executive Mixed vs singles score** |  |  |  |
| Mean (SD) | 0.1 (2.74) | 0.3 (2.56) | 0.2 (2.66) |
| OCS impairment (score>4), n/N (%) | 36/319 (11.3%) | 22/257 (8.6%) | 58/576 (10.1%) |

**Figure-S1 Forest plot of predictors of missing primary outcome data at 12 months***

|  |  |
| --- | --- |
| *Predictors of missing primary outcome data were conducted using logistic regression with dependent variable primary outcome availability and independent variables for treatment, baseline characteristic and their interaction. Odds Ratios (OR) of primary outcome availability are depicted in grey where there was no evidence, and in blue where there was at least some evidence (main effect p<0.1) of an overall association between the baseline characteristic and missingness (irrespective of arm) and represent the OR in the UC arm. ORs are included in yellow for the ESSVR arm where there was at least some evidence (Treatment interaction p<0.1) of a differential association by arm. | |

**Figure-S2 Forest plot of predictors of missing full questionnaire outcome data at 12 months***

| **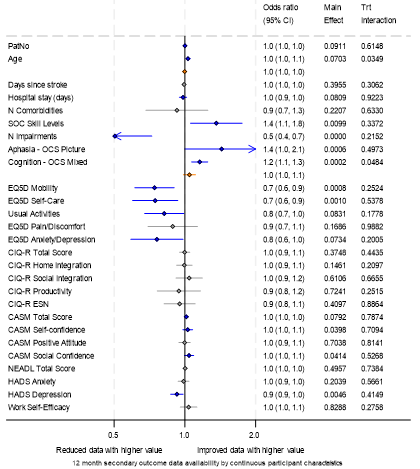** | **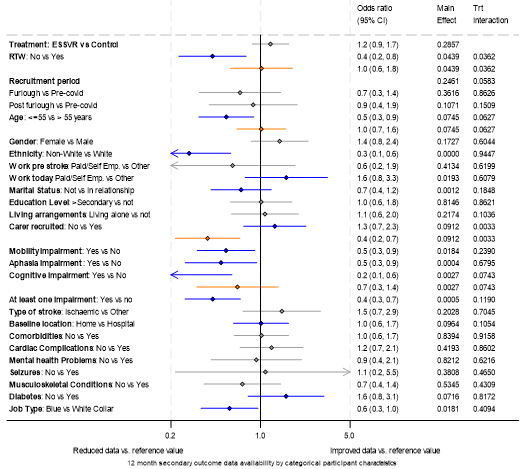** |
| --- | --- |
| *Predictors of missing full questionnaire outcome data were conducted using logistic regression with dependent variable questionnaire (at least partially completed) availability and independent variables for treatment, baseline characteristic and their interaction. Odds Ratios (OR) of primary outcome availability are depicted in grey where there was no evidence, and in blue where there was at least some evidence (main effect p<0.1) of an overall association between the baseline characteristic and missingness (irrespective of arm) and represent the OR in the UC arm. ORs are included in yellow for the ESSVR arm where there was at least some evidence (Treatment interaction p<0.1) of a differential association by arm. | |

**Table-S4 Study conduct^[[5]](#footnote-5)^**

|  | **ESSVR (n=324)** | **UC (n=259)** | **Total (n=583)** |
| --- | --- | --- | --- |
| **Eligibility violator?** | 2 (0.6%) | 2 (0.8%) | 4 (0.7%) |
|  |  |  |  |
| Contamination – UC participant seen by RETAKE OT | NA | 4 (1.5%) | NA |
|  |  |  |  |
| **Researcher unblinded** | 18 (5.6%) | 10 (3.9%) | 28 (4.8%) |
|  |  |  |  |
| **Participant withdrawal** | 18 (5.6%) | 17 (6.6%) | 35 (6.0%) |
| Questionnaires | 13 (4.0%) | 11 (4.2%) | 24 (4.1%) |
| Receipt of SMS text | 12 (3.7%) | 14 (5.4%) | 26 (4.5%) |
| Process evaluation | 13 (4.0%) | 8 (3.1%) | 21 (3.6%) |
| Access to records / routine data | 13 (4.0%) | 7 (2.7%) | 20 (3.4%) |
|  |  |  |  |
| Participant died (within 12 months) | 1 (0.3%) | 4 (1.5%) | 5 (0.9%) |

**Table-S5 Primary and secondary outcome effect estimates (and 95% confidence intervals) from primary and sensitivity analysis^[[6]](#footnote-6)^**

|  | **3 months** | | | **6 months** | | | **12 months** | | |
| --- | --- | --- | --- | --- | --- | --- | --- | --- | --- |
|  | **Sensitivity analyses** | | **Primary multiply imputed analysis** | **Sensitivity analyses** | | **Primary multiply imputed analysis** | **Sensitivity analyses** | | **Primary multiply imputed analysis** |
|  | **Complete case, no OT RE** | **Complete case** |  | **Complete case, no OT RE** | **Complete case** |  | **Complete case, no OT RE** | **Complete case** |  |
| **PRIMARY OUTCOME**  **Return to work** | 1.11 ( 0.69, 1.78), p=0.6678 | 1.11 ( 0.68, 1.82), p=0.6708 | 1.02 (0.65, 1.60), p=0.9283 | 1.00 ( 0.61, 1.63), p=0.9917 | 1.00 ( 0.60, 1.67), p=0.9904 | 1.00 (0.65, 1.52), p=0.9884 | 1.22 ( 0.80, 1.87), p=0.3582 | 1.22 ( 0.79, 1.90), p=0.3698 | 1.12 (0.75, 1.68), p=0.5678 |
|  |  |  |  |  |  |  |  |  |  |
| **SECONDARY OUTCOMES** |  |  |  |  |  |  |  |  |  |
| **Mood: HADs-Anxiety^[[7]](#footnote-7)^** | 0.18 (-0.73, 1.10), p=0.6924 | 0.17 (-0.80, 1.13), p=0.7341 | 0.43 (-0.48, 1.34), p=0.3518 | -0.05 (-0.99, 0.89), p=0.9106 | -0.06 (-0.99, 0.87), p=0.8996 | 0.60 (-0.32, 1.53), p=0.2000 | -0.21 (-1.28, 0.87), p=0.7049 | -0.20 (-1.24, 0.85), p=0.7137 | 0.24 (-0.71, 1.20), p=0.6174 |
| **Mood: HADs-Depression^2^** | 0.21 (-0.69, 1.11), p=0.6417 | 0.23 (-0.66, 1.12), p=0.6113 | 0.40 (-0.49, 1.29), p=0.3772 | 0.12 (-0.77, 1.00), p=0.7950 | 0.13 (-0.78, 1.04), p=0.7781 | 0.56 (-0.36, 1.48), p=0.2305 | -0.15 (-1.19, 0.89), p=0.7756 | -0.15 (-1.19, 0.89), p=0.7737 | 0.58 (-0.40, 1.56), p=0.2416 |
| **Functional ability: NEADL^[[8]](#footnote-8)^** | NA | NA | NA | 0.22 (-2.37, 2.80), p=0.8694 | 0.20 (-2.44, 2.83), p=0.8833 | -1.05 (-3.96, 1.86), p=0.4755 | -1.48 (-4.28, 1.33), p=0.3003 | -1.48 (-4.19, 1.23), p=0.2820 | **-3.37 (-6.26, -0.48), p=0.0230*** |
| **Participation: CIQ-R Social Integration^[[9]](#footnote-9)^** | NA | NA | NA | NA | NA | NA | -0.28 (-0.80, 0.24), p=0.2948 | -0.28 (-0.80, 0.24), p=0.2949 | -0.36 (-0.86, 0.13), p=0.1493 |
| **Participation: CIQ-R Productivity^4^** | NA | NA | NA | NA | NA | NA | -0.09 (-0.60, 0.43), p=0.7441 | -0.08 (-0.60, 0.44), p=0.7627 | -0.40 (-0.82, 0.01), p=0.0571 |
| **Self-efficacy: WAI^[[10]](#footnote-10)^** | -0.16 (-0.81, 0.50), p=0.6377 | -0.19 (-0.89, 0.50), p=0.5799 | -0.44 (-1.06, 0.17), p=0.1551 | 0.01 (-0.61, 0.64), p=0.9664 | 0.01 (-0.65, 0.68), p=0.9671 | -0.27 (-0.84, 0.30), p=0.3537 | -0.16 (-0.87, 0.55), p=0.6630 | -0.17 (-0.90, 0.55), p=0.6360 | -0.45 (-1.18, 0.28), p=0.2226 |
| **Post-stroke confidence: CASM^[[11]](#footnote-11)^** | NA | NA | NA | NA | NA | NA | 1.65 (-1.78, 5.08), p=0.3428 | 1.62 (-1.91, 5.16), p=0.3625 | -0.79 (-3.64, 2.06), p=0.5837 |
| **Carer burden: MSCI^[[12]](#footnote-12)^** | -0.20 (-2.52, 2.11), p=0.8595 | -0.33 (-2.50, 1.85), p=0.7620 | -0.27 (-2.08, 1.54), p=0.7681 | -0.57 (-4.52, 3.38), p=0.7700 | -0.63 (-4.61, 3.34), p=0.7434 | 0.87 (-1.59, 3.32), p=0.4858 | 0.77 (-1.88, 3.42), p=0.5574 | 0.77 (-1.91, 3.46), p=0.5575 | **2.52 (0.63, 4.41), p=0.0095*** |

**Figure-S3 Adjusted mean scores and 95%CIs estimated from multiply imputed adjusted analyses of secondary outcomes^[[13]](#footnote-13)^**

| 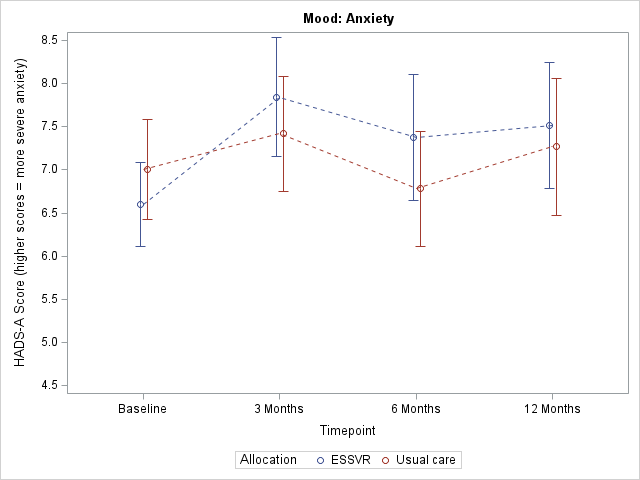 | 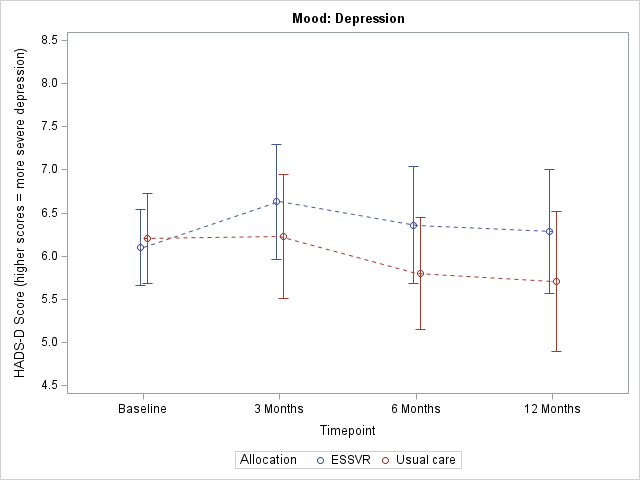 |
| --- | --- |
| 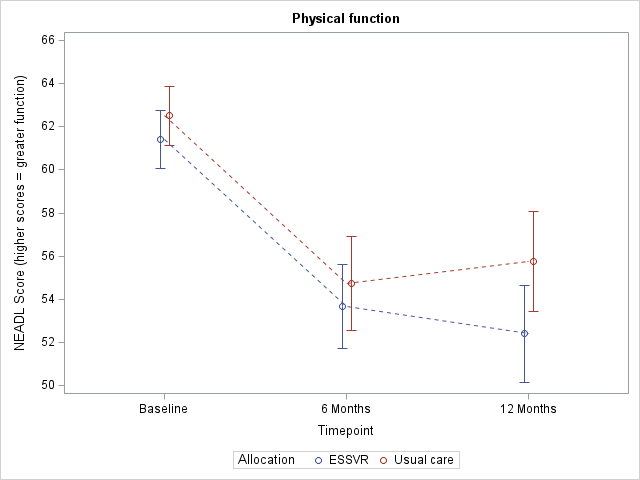 | 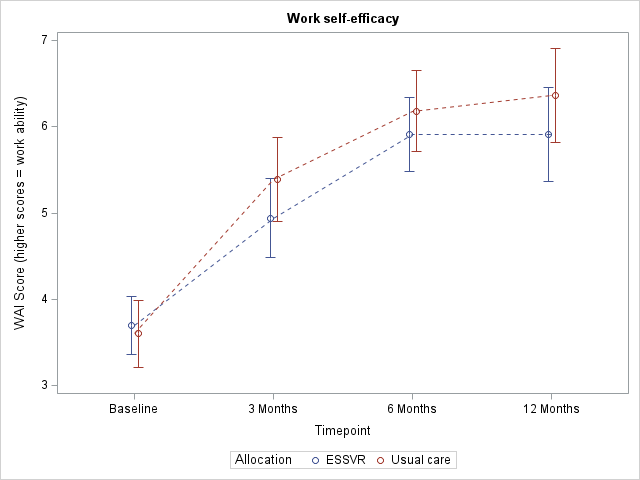 |
| 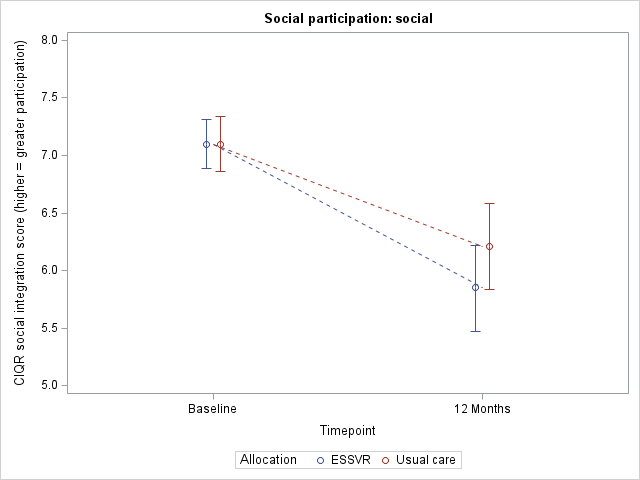 | 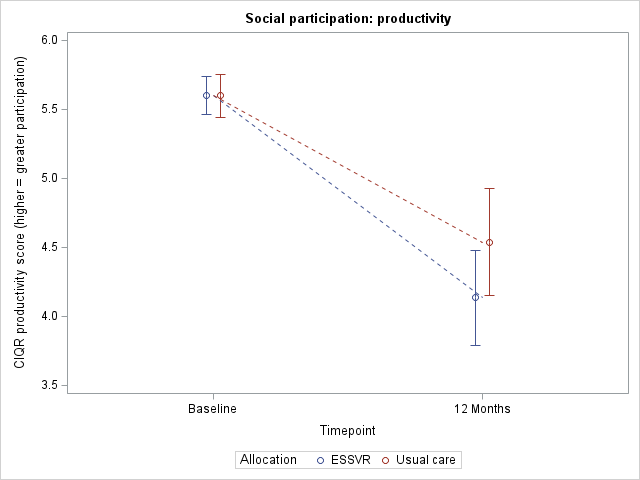 |
| 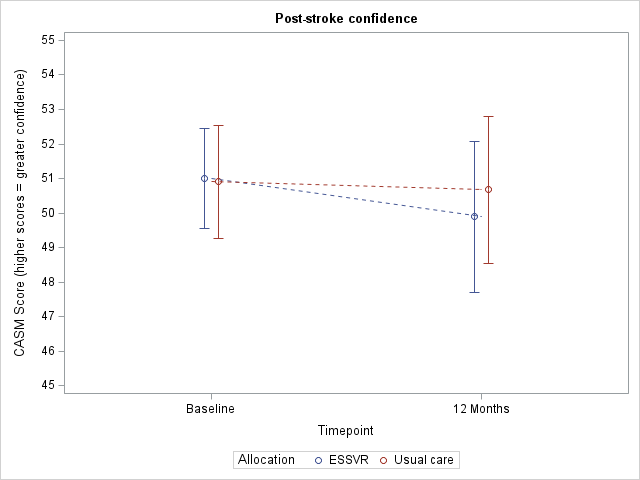 | 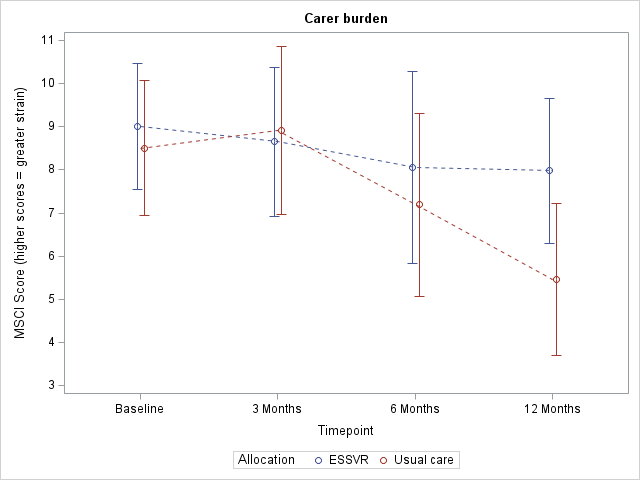 |

**Table-S6 Baseline covariates by 12-month RTW Primary outcome^[[14]](#footnote-14)^**

|  | **12-month RTW Primary Outcome** | | |  |
| --- | --- | --- | --- | --- |
|  | **Missing (n=129)** | **Yes (n=282)** | **No (n=172)** | **Total (n=583)** |
| **Randomisation allocation** |  |  |  |  |
| ESSVR | 67 (51.9%) | 165 (58.5%) | 92 (53.5%) | 324 (55.6%) |
| UC only | 62 (48.1%) | 117 (41.5%) | 80 (46.5%) | 259 (44.4%) |
| **Age** |  |  |  |  |
| Mean (s.d.) | 53.6 (11.66) | 52.9 (10.34) | 56.1 (11.71) | 54.0 (11.12) |
| Median (range) | 55.0 (19.0, 82.0) | 54.0 (20.0, 78.0) | 57.0 (26.0, 93.0) | 55.0 (19.0, 93.0) |
| IQR | 48.0, 61.0 | 47.0, 60.0 | 50.0, 62.0 | 48.0, 61.0 |
| Missing | 0 | 0 | 0 | 0 |
| **Gender** |  |  |  |  |
| Male | 87 (69.0%) | 195 (69.1%) | 118 (68.6%) | 400 (69.0%) |
| Female | 39 (31.0%) | 87 (30.9%) | 54 (31.4%) | 180 (31.0%) |
| Missing | 3 | 0 | 0 | 3 |
| **Mobility (EQ-5D-5L Mobility Score^[[15]](#footnote-15)^)** |  |  |  |  |
| Mean (s.d.) | 2.5 (1.40) | 2.0 (1.15) | 2.6 (1.32) | 2.3 (1.29) |
| Median (range) | 2.0 (1.0, 5.0) | 2.0 (1.0, 5.0) | 2.0 (1.0, 5.0) | 2.0 (1.0, 5.0) |
| IQR | 1.0, 3.0 | 1.0, 3.0 | 2.0, 3.5 | 1.0, 3.0 |
| Missing | 0 | 0 | 0 | 0 |
| **Aphasia (OCS Picture Naming Score^[[16]](#footnote-16)^)** |  |  |  |  |
| Mean (s.d.) | 3.5 (0.94) | 3.8 (0.57) | 3.7 (0.74) | 3.7 (0.72) |
| Median (range) | 4.0 (0.0, 4.0) | 4.0 (0.0, 4.0) | 4.0 (0.0, 4.0) | 4.0 (0.0, 4.0) |
| IQR | 3.0, 4.0 | 4.0, 4.0 | 4.0, 4.0 | 4.0, 4.0 |
| Missing | 0 | 0 | 0 | 0 |
| **Cognition (OCS Mixed Score^[[17]](#footnote-17)^)** |  |  |  |  |
| Mean (s.d.) | 10.2 (4.33) | 11.8 (2.75) | 10.4 (3.93) | 11.0 (3.59) |
| Median (range) | 13.0 (0.0, 13.0) | 13.0 (0.0, 13.0) | 13.0 (0.0, 13.0) | 13.0 (0.0, 13.0) |
| IQR | 8.0, 13.0 | 12.0, 13.0 | 8.0, 13.0 | 11.0, 13.0 |
| Missing | 0 | 0 | 0 | 0 |

**Table-S7 Work status related to COVID-19**

|  | **3 months** | | | **6 months** | | | **12 months** | | |
| --- | --- | --- | --- | --- | --- | --- | --- | --- | --- |
|  | **ESSVR (n=324)** | **UC (n=259)** | **Total (n=583)** | **ESSVR (n=324)** | **UC (n=259)** | **Total (n=583)** | **ESSVR (n=324)** | **UC (n=259)** | **Total (n=583)** |
| **N (%) participants with data available** | 1 (0.3%) | 2 (0.8%) | 3 (0.5%) | 18 (5.6%) | 13 (5.0%) | 31 (5.3%) | 29 (9.0%) | 34 (13.1%) | 63 (10.8%) |
| **Participant in work prior to COVID-19** |  |  |  |  |  |  |  |  |  |
| Yes | 1 (100.0%) | 2 (100.0%) | 3 (100.0%) | 13 (72.2%) | 9 (69.2%) | 22 (71.0%) | 23 (79.3%) | 24 (70.6%) | 47 (74.6%) |
| No | 0 (0.0%) | 0 (0.0%) | 0 (0.0%) | 5 (27.8%) | 4 (30.8%) | 9 (29.0%) | 6 (20.7%) | 10 (29.4%) | 16 (25.4%) |
| **If yes, work status changed due to COVID-19** |  |  |  |  |  |  |  |  |  |
| Yes | 0 (0.0%) | 0 (0.0%) | 0 (0.0%) | 5 (38.5%) | 4 (44.4%) | 9 (40.9%) | 12 (52.2%) | 9 (37.5%) | 21 (44.7%) |
| No | 1 (100.0%) | 2 (100.0%) | 3 (100.0%) | 8 (61.5%) | 5 (55.6%) | 13 (59.1%) | 11 (47.8%) | 15 (62.5%) | 26 (55.3%) |
| **If yes, current work status** |  |  |  |  |  |  |  |  |  |
| Not working |  |  |  | 2 (50.0%) | 2 (50.0%) | 4 (50.0%) | 6 (50.0%) | 6 (66.7%) | 12 (57.1%) |
| Returned to work |  |  |  | 2 (50.0%) | 2 (50.0%) | 4 (50.0%) | 6 (50.0%) | 3 (33.3%) | 9 (42.9%) |
| Missing |  |  |  | 1 | 0 | 1 | 0 | 0 | 0 |
| **Reason not working** |  |  |  |  |  |  |  |  |  |
| Self-isolating |  |  |  | 0 (0.0%) | 0 (0.0%) | 0 (0.0%) | 0 (0.0%) | 1 (20.0%) | 1 (9.1%) |
| Childcare / caring responsibilities |  |  |  | 0 (0.0%) | 0 (0.0%) | 0 (0.0%) | 0 (0.0%) | 0 (0.0%) | 0 (0.0%) |
| Furloughed |  |  |  | 2 (100.0%) | 0 (0.0%) | 2 (50.0%) | 2 (33.3%) | 1 (20.0%) | 3 (27.3%) |
| Made redundant |  |  |  | 0 (0.0%) | 1 (50.0%) | 1 (25.0%) | 2 (33.3%) | 2 (40.0%) | 4 (36.4%) |
| Self-employed and unable to work |  |  |  | 0 (0.0%) | 1 (50.0%) | 1 (25.0%) | 1 (16.7%) | 0 (0.0%) | 1 (9.1%) |
| Due to return to work but unable |  |  |  | 0 (0.0%) | 0 (0.0%) | 0 (0.0%) | 1 (16.7%) | 0 (0.0%) | 1 (9.1%) |
| Other^[[18]](#footnote-18)^ |  |  |  | 0 (0.0%) | 0 (0.0%) | 0 (0.0%) | 0 (0.0%) | 1 (20.0%) | 1 (9.1%) |
| Missing |  |  |  | 0 | 0 | 0 | 0 | 1 | 1 |
| **Returned to work status^[[19]](#footnote-19)^** |  |  |  |  |  |  |  |  |  |
| Returned to full hours with same employer |  |  |  | 2 (100.0%) | 1 (50.0%) | 3 (75.0%) | 0 (0.0%) | 0 (0.0%) | 0 (0.0%) |
| Returned to work but on reduced hours |  |  |  | 0 (0.0%) | 0 (0.0%) | 0 (0.0%) | 4 (66.7%) | 1 (33.3%) | 5 (55.6%) |
| Other^[[20]](#footnote-20)^ |  |  |  | 0 (0.0%) | 1 (50.0%) | 1 (25.0%) | 2 (33.3%) | 2 (66.7%) | 4 (44.4%) |
| **If not working due to COVID-19, is this:** |  |  |  |  |  |  |  |  |  |
| Unsure |  |  |  | 1 (50.0%) | 2 (100.0%) | 3 (75.0%) | 2 (33.3%) | 3 (50.0%) | 5 (41.7%) |
| Changes in job status are permanent |  |  |  | 0 (0.0%) | 0 (0.0%) | 0 (0.0%) | 0 (0.0%) | 1 (16.7%) | 1 (8.3%) |
| Plan to return to work once government restrictions are lifted |  |  |  | 1 (50.0%) | 0 (0.0%) | 1 (25.0%) | 4 (66.7%) | 2 (33.3%) | 6 (50.0%) |

**Table-S8 Other COVID-19 Impacts**

|  | **3 months** | | | **6 months** | | | **12 months** | | |
| --- | --- | --- | --- | --- | --- | --- | --- | --- | --- |
|  | **ESSVR (n=324)** | **UC (n=259)** | **Total (n=583)** | **ESSVR (n=324)** | **UC (n=259)** | **Total (n=583)** | **ESSVR (n=324)** | **UC (n=259)** | **Total (n=583)** |
| **N (%) participants with data available** | 1 (0.3%) | 2 (0.8%) | 3 (0.5%) | 18 (5.6%) | 13 (5.0%) | 31 (5.3%) | 29 (9.0%) | 34 (13.1%) | 63 (10.8%) |
| **Have you had COVID-19** |  |  |  |  |  |  |  |  |  |
| Unsure | 0 (0.0%) | 0 (0.0%) | 0 (0.0%) | 0 (0.0%) | 1 (7.7%) | 1 (3.2%) | 0 (0.0%) | 1 (2.9%) | 1 (1.6%) |
| Yes, diagnosed and recovered | 1 (100.0%) | 0 (0.0%) | 1 (33.3%) | 2 (11.1%) | 1 (7.7%) | 3 (9.7%) | 1 (3.4%) | 0 (0.0%) | 1 (1.6%) |
| Yes, diagnosed and still ill | 0 (0.0%) | 0 (0.0%) | 0 (0.0%) | 0 (0.0%) | 0 (0.0%) | 0 (0.0%) | 0 (0.0%) | 0 (0.0%) | 0 (0.0%) |
| Not formally diagnosed but suspected | 0 (0.0%) | 0 (0.0%) | 0 (0.0%) | 0 (0.0%) | 1 (7.7%) | 1 (3.2%) | 1 (3.4%) | 1 (2.9%) | 2 (3.2%) |
| No | 0 (0.0%) | 2 (100.0%) | 2 (66.7%) | 16 (88.9%) | 10 (76.9%) | 26 (83.9%) | 27 (93.1%) | 32 (94.1%) | 59 (93.7%) |
| **What is your current situation** |  |  |  |  |  |  |  |  |  |
| I am living my life as normal | 0 (0.0%) | 1 (50.0%) | 1 (33.3%) | 2 (11.1%) | 4 (30.8%) | 6 (19.4%) | 7 (24.1%) | 9 (26.5%) | 16 (25.4%) |
| I am social distancing | 1 (100.0%) | 1 (50.0%) | 2 (66.7%) | 16 (88.9%) | 9 (69.2%) | 25 (80.6%) | 21 (72.4%) | 21 (61.8%) | 42 (66.7%) |
| I am self-isolating | 0 (0.0%) | 0 (0.0%) | 0 (0.0%) | 0 (0.0%) | 0 (0.0%) | 0 (0.0%) | 1 (3.4%) | 4 (11.8%) | 5 (7.9%) |
| **Have you experienced any of the following** |  |  |  |  |  |  |  |  |  |
| Unable to pay bills/rent/mortgage | 0 (0.0%) | 0 (0.0%) | 0 (0.0%) | 1 (5.6%) | 1 (8.3%) | 2 (6.7%) | 0 (0.0%) | 0 (0.0%) | 0 (0.0%) |
| Unable to access sufficient food | 0 (0.0%) | 0 (0.0%) | 0 (0.0%) | 1 (5.6%) | 0 (0.0%) | 1 (3.3%) | 0 (0.0%) | 0 (0.0%) | 0 (0.0%) |
| Unable to access required medication | 0 (0.0%) | 0 (0.0%) | 0 (0.0%) | 1 (5.6%) | 0 (0.0%) | 1 (3.3%) | 0 (0.0%) | 0 (0.0%) | 0 (0.0%) |
| You have been ill in hospital (due to COVID-19) | 0 (0.0%) | 0 (0.0%) | 0 (0.0%) | 1 (5.6%) | 0 (0.0%) | 1 (3.3%) | 0 (0.0%) | 0 (0.0%) | 0 (0.0%) |
| Someone close to you has been ill in hospital (due to COVID-19 or another illness) | 0 (0.0%) | 0 (0.0%) | 0 (0.0%) | 2 (11.1%) | 1 (8.3%) | 3 (10.0%) | 0 (0.0%) | 0 (0.0%) | 0 (0.0%) |
| You have lost someone close to you (due to COVID-19) | 0 (0.0%) | 0 (0.0%) | 0 (0.0%) | 0 (0.0%) | 1 (8.3%) | 1 (3.3%) | 1 (3.4%) | 0 (0.0%) | 1 (1.6%) |
| Lost your job(s)/been unable to do paid work | 0 (0.0%) | 0 (0.0%) | 0 (0.0%) | 2 (11.1%) | 1 (8.3%) | 3 (10.0%) | 1 (3.4%) | 1 (3.0%) | 2 (3.2%) |
| Cut in household income | 0 (0.0%) | 0 (0.0%) | 0 (0.0%) | 2 (11.1%) | 1 (8.3%) | 3 (10.0%) | 2 (6.9%) | 1 (3.0%) | 3 (4.8%) |
| None of the above | 1 (100.0%) | 2 (100.0%) | 3 (100.0%) | 7 (38.9%) | 7 (58.3%) | 14 (46.7%) | 24 (82.8%) | 31 (93.9%) | 55 (88.7%) |
| Other^[[21]](#footnote-21)^ | 0 (0.0%) | 0 (0.0%) | 0 (0.0%) | 1 (5.6%) | 0 (0.0%) | 1 (3.3%) | 1 (3.4%) | 0 (0.0%) | 1 (1.6%) |
| Missing | 0 | 0 | 0 | 0 | 1 | 1 | 0 | 1 | 1 |
| **Have you applied for/received any financial help** |  |  |  |  |  |  |  |  |  |
| Yes | 0 (0.0%) | 0 (0.0%) | 0 (0.0%) | 0 (0.0%) | 1 (7.7%) | 1 (3.2%) | 8 (27.6%) | 7 (21.2%) | 15 (24.2%) |
| No | 1 (100.0%) | 2 (100.0%) | 3 (100.0%) | 18 (100.0%) | 12 (92.3%) | 30 (96.8%) | 21 (72.4%) | 26 (78.8%) | 47 (75.8%) |
| Missing | 0 | 0 | 0 | 0 | 0 | 0 | 0 | 1 | 1 |
| **If yes, applied/received (not mutually exclusive)^[[22]](#footnote-22)^** |  |  |  |  |  |  |  |  |  |
| Universal credit |  |  |  | 0 (0.0%) | 0 (0.0%) | 0 (0.0%) | 1 (12.5%) | 1 (14.3%) | 2 (13.3%) |
| Government grant^[[23]](#footnote-23)^ |  |  |  | 0 (0.0%) | 0 (0.0%) | 0 (0.0%) | 6 (75.0%) | 6 (85.7%) | 12 (80.0%) |
| Other |  |  |  | 0 (0.0%) | 0 (0.0%) | 0 (0.0%) | 1 (12.5%) | 0 (0.0%) | 1 (6.7%) |

**Table-S9 12-month secondary outcomes by primary RTW outcome overall and by arm^[[24]](#footnote-24)^**

|  | **RTW Primary Outcome: Total** | | | **RTW Primary Outcome: UC** | | | **RTW Primary Outcome: ESSVR** | | |
| --- | --- | --- | --- | --- | --- | --- | --- | --- | --- |
|  | **Yes**  **N=282** | **No**  **N=172** | **Total**  **N=454** | **Yes**  **N=117** | **No**  **N=80** | **Total**  **N=197** | **Yes**  **N=165** | **No**  **N=92** | **Total**  **N=257** |
| **Mood: HADs-Anxiety^[[25]](#footnote-25)^** |  |  |  |  |  |  |  |  |  |
| Mean (SD) | 6.5 (4.54) | 8.1 (5.31) | 7.0 (4.83) | 7.3 (4.22) | 7.3 (5.48) | 7.2 (4.56) | 5.9 (4.70) | 8.5 (5.21) | 6.8 (5.01) |
| N | 175 | 82 | 259 | 72 | 30 | 104 | 103 | 52 | 155 |
| **Mood: HADs-Depression^2^** |  |  |  |  |  |  |  |  |  |
| Mean (SD) | 4.6 (4.03) | 7.5 (4.46) | 5.6 (4.41) | 4.9 (4.07) | 6.5 (4.06) | 5.4 (4.13) | 4.3 (4.01) | 8.1 (4.60) | 5.7 (4.59) |
| N | 177 | 83 | 263 | 73 | 30 | 105 | 104 | 53 | 158 |
| **Functional ability: NEADL^[[26]](#footnote-26)^** |  |  |  |  |  |  |  |  |  |
| Mean (SD) | 59.6 (8.11) | 47.8 (15.35) | 55.8 (12.36) | 60.7 (7.82) | 51.2 (13.71) | 57.9 (10.75) | 58.9 (8.26) | 45.7 (16.04) | 54.3 (13.20) |
| N | 179 | 84 | 266 | 75 | 32 | 109 | 104 | 52 | 157 |
| **Participation: CIQ-R Social Integration^[[27]](#footnote-27)^** |  |  |  |  |  |  |  |  |  |
| Mean (SD) | 6.5 (2.04) | 5.7 (2.45) | 6.2 (2.21) | 6.7 (2.07) | 6.1 (2.39) | 6.5 (2.16) | 6.4 (2.02) | 5.4 (2.46) | 6.0 (2.24) |
| N | 174 | 85 | 262 | 74 | 33 | 109 | 100 | 52 | 153 |
| **Participation: CIQ-R Productivity^4^** |  |  |  |  |  |  |  |  |  |
| Mean (SD) | 5.5 (1.07) | 2.1 (1.56) | 4.4 (2.04) | 5.6 (1.17) | 2.2 (1.54) | 4.6 (2.03) | 5.5 (0.98) | 2.0 (1.59) | 4.3 (2.04) |
| N | 172 | 81 | 255 | 73 | 32 | 106 | 99 | 49 | 149 |
| **Work self-efficacy: WAI^[[28]](#footnote-28)^** |  |  |  |  |  |  |  |  |  |
| Mean (SD) | 7.7 (1.77) | 3.7 (3.07) | 6.4 (2.97) | 7.6 (1.77) | 4.5 (3.41) | 6.6 (2.82) | 7.8 (1.77) | 3.1 (2.73) | 6.2 (3.08) |
| N | 177 | 86 | 265 | 75 | 34 | 111 | 102 | 52 | 154 |
| **Post-stroke confidence: CASM^[[29]](#footnote-29)^** |  |  |  |  |  |  |  |  |  |
| Mean (SD) | 55.0 (14.41) | 44.7 (12.87) | 51.5 (14.79) | 53.3 (14.03) | 49.4 (13.64) | 52.0 (13.89) | 56.3 (14.63) | 41.8 (11.58) | 51.2 (15.42) |
| N | 169 | 81 | 253 | 71 | 31 | 104 | 98 | 50 | 149 |
| **Carer burden: MSCI^[[30]](#footnote-30)^** |  |  |  |  |  |  |  |  |  |
| Mean (SD) | 6.3 (5.64) | 7.3 (5.97) | 6.8 (5.88) | 4.3 (4.79) | 3.1 (3.67) | 3.9 (4.31) | 7.5 (5.86) | 8.4 (6.07) | 8.1 (6.08) |
| N | 33 | 19 | 54 | 12 | 4 | 17 | 21 | 15 | 37 |

This table presents an exploratory comparison of secondary outcomes by participants primary outcome return-to-work status and shows participants who returned to work had lower levels of anxiety and depression, and were more functionally independent, confident, and socially integrated and had greater work self-efficacy than those who did not.

**Figure-S4 Moderator analysis: Odds Ratio and predicted probability (95%CI) of 12-month return-to-work by treatment according to moderators age and number of impairments**

| **Age** | **Number of Impairments** |
| --- | --- |
|  | 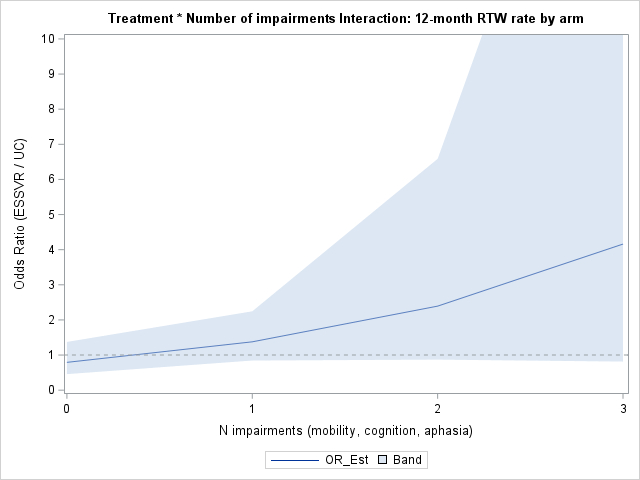 |
| 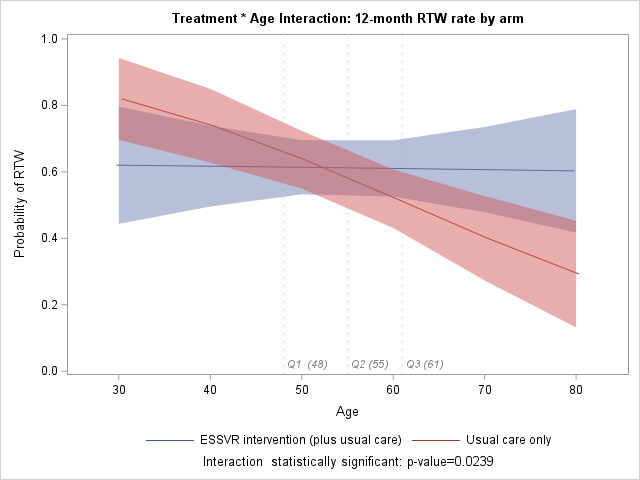 | 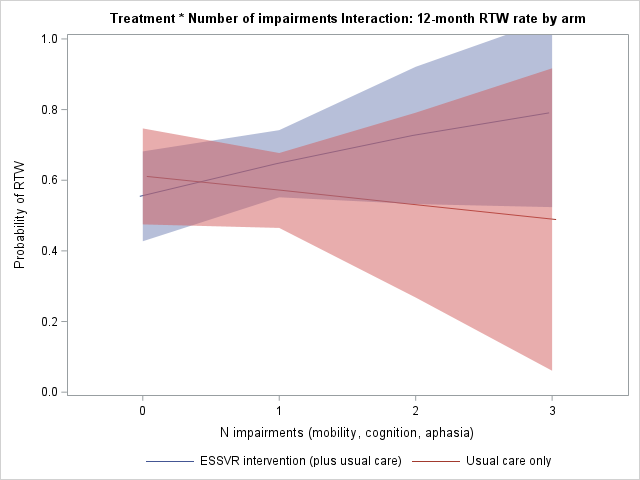 |

**Table-S10 Death and safety outcomes^[[31]](#footnote-31)^***

|  | **3 months** | | | **6 months** | | | **12 months** | | |
| --- | --- | --- | --- | --- | --- | --- | --- | --- | --- |
|  | **ESSVR**  **(n=324)** | **UC**  **(n=259)** | **Total**  **(n=583)** | **ESSVR**  **(n=324)** | **UC**  **(n=259)** | **Total**  **(n=583)** | **ESSVR**  **(n=324)** | **UC**  **(n=259)** | **Total**  **(n=583)** |
| **Questionnaire returned** | 195 (60.2%) | 144 (55.6%) | 339 (58.1%) | 199 (61.4%) | 142 (54.8%) | 341 (58.5%) | 182 (56.2%) | 134 (51.7%) | 316 (54.2%) |
|  |  |  |  |  |  |  |  |  |  |
| **SAFETY OUTCOMES** |  |  |  |  |  |  |  |  |  |
| **Participant died** |  |  |  |  |  |  | 1/324 (0.3%) | 4/259 (1.5%) | 5/583 (0.9%) |
| **Attended A&E?** | 27/174 (15.5%) | 12/118 (10.2%) | 39/292 (13.4%) | 16/169 (9.5%) | 14/119 (11.8%) | 30/288 (10.4%) | 23/140 (16.4%) | 17/101 (16.8%) | 40/241 (16.6%) |
| **If yes, N attendances, mean(SD)** | 1.5 (0.96) | 1.5 (0.71) | 1.5 (0.89) | 1.3 (0.90) | 1.5 (0.69) | 1.4 (0.81) | 1.7 (1.53) | 1.5 (0.83) | 1.6 (1.25) |
|  |  |  |  |  |  |  |  |  |  |
| **Admitted to hospital?** | 30/181 (16.6%) | 19/129 (14.7%) | 49/310 (15.8%) | 13/182 (7.1%) | 10/130 (7.7%) | 23/312 (7.4%) | 15/156 (9.6%) | 9/108 (8.3%) | 24/264 (9.1%) |
| **If yes, N admissions, mean(SD)** | 1.4 (0.84) | 1.9 (1.08) | 1.6 (0.97) | 1.2 (0.60) | 1.6 (0.70) | 1.4 (0.66) | 1.1 (0.26) | 1.2 (0.44) | 1.1 (0.34) |
|  |  |  |  |  |  |  |  |  |  |
| **Work accident?** | 2/183 (1.1%) | 0/127 (0.0%) | 2/310 (0.6%) | 4/181 (2.2%) | 1/129 (0.8%) | 5/310 (1.6%) | 4/159 (2.5%) | 2/107 (1.9%) | 6/266 (2.3%) |
|  |  |  |  |  |  |  |  |  |  |

**REFERENCES**

1. Insights P. What is driving the Great Retirement? 2022 [Available from: <https://www.thephoenixgroup.com/phoenix-insights/publications/what-driving-great-retirement/>.

2. Radford KA, Wright-Hughes A, Clarke D, et al. RETurn to work After stroKE (RETAKE) 15/130/11. Synopsis submitted to Health Technology Assessment

3. Trusson D, Powers K, Radford KA, et al. Exploring stroke survivor and employer experiences of return-to-work support within the RETurn to work After stroKE (RETAKE) trial during the COVID-19 pandemic. submitted to Frontiers in Sociology.

4. Santomauro DF, Herrera AMM, Shadid J, et al. Global prevalence and burden of depressive and anxiety disorders in 204 countries and territories in 2020 due to the COVID-19 pandemic. The Lancet. 2021;398(10312):1700-12.

5. Organization WH. Mental health and COVID-19: early evidence of the pandemic’s impact: scientific brief, 2 March 2022. World Health Organization; 2022.

6. Association BM. Delivery of healthcare during the pandemic BMA Covid Review 3. 2022.

7. Abdul Rashid MR, Syed Mohamad SN, Tajjudin AIA, et al. COVID-19 pandemic fatigue and its sociodemographic, mental health status, and perceived causes: a cross-sectional study nearing the transition to an endemic phase in Malaysia. International journal of environmental research and public health. 2023;20(5):4476.

8. Feigin VL, Stark BA, Johnson CO, et al. Global, regional, and national burden of stroke and its risk factors, 1990–2019: a systematic analysis for the Global Burden of Disease Study 2019. The Lancet Neurology. 2021;20(10):795-820.

9. Skivington K, Matthews L, Simpson SA, et al. A new framework for developing and evaluating complex interventions: update of Medical Research Council guidance. bmj. 2021;374.

10. (ONS) OfNS. Rising ill-health and economic inactivity because of long-term sickness, UK: 2019 to 2023.

11. England N. Stroke Vocational Rehabilitation Toolkit [Available from: <https://www.e-lfh.org.uk/programmes/strokevrtoolkit/>.

12. England N. Inclusive digital healthcare: a framework for NHS action on digital inclusion. 2023.

13. Impact assessment. Coronavirus Job Retention Scheme. 2022.

14. Marmot M. Health equity in England: the Marmot review 10 years on. Bmj. 2020;368.

15. Butink M, Boekel L, Boonen A, et al. Work participation and the COVID-19 pandemic: an observational study in people with inflammatory rheumatic diseases and population controls. Rheumatology advances in practice. 2024;8(2):rkae026.

16. De Dios Perez B, Pritchard C, Powers K, et al. The Impact of COVID-19 on the Employment of People With Multiple Sclerosis: A Multi-Methods Study. International Journal of MS Care. 2024.

17. (NICE). NIfHaCE. Stroke Rehabilitation in Adults NICE guideline. . wwwniceorguk/guidance/ng2362023.

18. Party ISW. National clinical guideline for stroke for the UK and Ireland, 2023 Edition. wwwstrokeguidelineorg. 2023.

19. Programme SSNA. Sentinel Stroke National Audit Programme (SSNAP) Post-acute Organisational Audit Report National Report, Section 7: Vocational rehabilitation.2021.

1. Mobility impairment=Eq-5D-5L moderate/severe problems in walking about/unable to walk. [↑](#footnote-ref-1)
2. Aphasia impairment=OCS picture naming task score ≤3/4 (≤5th centile of normative data indicating impairment on expressive language). [↑](#footnote-ref-2)
3. Cognitive impairment= OCS executive mixed task scores ≤4/13 (≤5th centile of normative data impairment on Task switching/Attention). [↑](#footnote-ref-3)
4. Cut offs are based on scores lower than the 5th centile from normative data[25] except for the picture naming and executive mixed scored which were defined based on scores lower than or equal to the 5th centile (to include milder levels of impairment) for RETAKE aphasia and cognitive impairment summaries. Impairment according to scores lower than the 5th centile gave 41 (7.0%) and 47 (8.1%) as impaired according to picture naming and executive mixed scores respectively.

   Higher scores indicate greater ability for all but the Executive Mixed vs singles score, Broken hearts – space and object asymmetry scores. Semantics and number writing scores range 0 to 3. Visual Field, calculation, verbal and episodic memory, and picture naming scores range 0 to 4. Sentence reading score ranges from 0 to 15. Broken hearts overall accuracy scores range from 0 to 50. Praxis scores range from 0 to 12. Executive mixed task scores range 0 to 13. [↑](#footnote-ref-4)
5. Eligibility violations occurred in four (<1%) participants (two per arm). Contamination was recorded for four (1.5%) UC participants who were seen at least once in UC by a RETAKE OT in error. Researchers were unblinded for 18/324 (5.6%) participants in ESSVR and 10/259 (3.9%) in UC. Withdrawals from questionnaires, receipt of SMS texts, process evaluation, and access to records/routine data, occurred in 18/324 (5.6%) participants in ESSVR and 17/259 (6.6%) in UC. Five (<1%) participants died within the 12-month follow-up period; one in ESSVR and four in UC. [↑](#footnote-ref-5)
6. Effects for the primary outcome represent the adjusted Odds Ratio (ESSVR/UC), and the adjusted mean difference (ESSVR – UC) for secondary outcomes. Estimates in **bold*** indicate statistically significant effects. [↑](#footnote-ref-6)
7. HADS-A and HADS-D scores range from 0 to 21, where higher scores indicate more severe anxiety and depression [↑](#footnote-ref-7)
8. NEADL score range from 0 to 66, where a higher score indicates greater functional ability [↑](#footnote-ref-8)
9. CIQ-R Social Integration scores range from 0 to 10 and Productivity scores 0 to 7 with higher scores indicating a greater degree of community integration. [↑](#footnote-ref-9)
10. WAI range from 0 to 10, where higher values indicate better work ability [↑](#footnote-ref-10)
11. CASM Score range from 0 to 81with higher scores indicating a higher level of confidence [↑](#footnote-ref-11)
12. MCSI is a carer rather than participant measure, n=71 and n=66 carers were recruited in the ESSVR and UC arms respectively. Missing data were imputed for recruited carers where applicable using a reduced imputation model based on covariates, outcome, and baseline values only. MCSI scores range from 0 to 26, where a higher score indicates a higher level of carer burden. [↑](#footnote-ref-12)
13. Baseline scores are the unadjusted mean (95%CI) [↑](#footnote-ref-13)
14. Excluding site [↑](#footnote-ref-14)
15. Eq-5D-5L Mobility scores range 1 to 5 with higher scores indicating greater mobility impairment/reduced mobility. [↑](#footnote-ref-15)
16. OCS picture naming task scores range 0 to 4 with higher scores indicating greater ability/reduced impairment on expressive language). [↑](#footnote-ref-16)
17. OCS executive mixed task range 0 to 13 with higher scores indicating greater ability/reduced impairment on Task switching/Attention). [↑](#footnote-ref-17)
18. Was volunteering at a school [↑](#footnote-ref-18)
19. No responses to: Returned to work but on altered duties, Returned to work sooner than planned, Not returned to pre-stroke work but started new temporary work, Not returned to pre-stroke work but started COVID-19 related volunteer work [↑](#footnote-ref-19)
20. Was unable to work due to lockdown restrictions, self employed was unable to work for a time, was furloughed but is now back at work x3 [↑](#footnote-ref-20)
21. Other details not provided [↑](#footnote-ref-21)
22. No responses as: Low interest loan, Deferment of tax payments, Welfare benefits [↑](#footnote-ref-22)
23. All government grants related to the furlough scheme, and one self-employed covid support grant [↑](#footnote-ref-23)
24. Column N are the number of participants with complete primary outcome data. [↑](#footnote-ref-24)
25. HADS-A and HADS-D scores range from 0 to 21, where higher scores indicate more severe anxiety and depression [↑](#footnote-ref-25)
26. NEADL score range from 0 to 66, where a higher score indicates greater functional ability [↑](#footnote-ref-26)
27. CIQ-R Social Integration scores range from 0 to 10 and Productivity scores 0 to 7 with higher scores indicating a greater degree of community integration. [↑](#footnote-ref-27)
28. WAI range from 0 to 10, where higher values indicate better work ability [↑](#footnote-ref-28)
29. CASM Score range from 0 to 81with higher scores indicating a higher level of confidence [↑](#footnote-ref-29)
30. MCSI is a carer rather than participant measure. MCSI scores range from 0 to 26, where a higher score indicates a higher level of carer burden [↑](#footnote-ref-30)
31. * There were no related unexpected serious adverse events. [↑](#footnote-ref-31)
